# PDGF regulates guanylate cyclase expression and cGMP signaling in vascular smooth muscle

**DOI:** 10.1038/s42003-022-03140-2

**Published:** 2022-03-03

**Authors:** Staffan Hildebrand, Mohamed Ibrahim, Andreas Schlitzer, Lars Maegdefessel, Wilhelm Röll, Alexander Pfeifer

**Affiliations:** 1 Institute of Pharmacology and Toxicology, University Hospital, University of Bonn, Bonn, Germany; 2 Quantitative Systems Biology, LIMES-Institute (Life and Medical Sciences Bonn), University of Bonn, Bonn, Germany; 3 Experimental Vascular Surgery and Medicine, Department of Vascular and Endovascular Surgery, Klinikum rechts der Isar - Technical University Munich, Munich, Germany; 4 Department of Medicine, Karolinska Institutet, Stockholm, Sweden; 5 Department of Cardiac Surgery, University of Bonn, Bonn, Germany

**Keywords:** Restenosis, Growth factor signalling

## Abstract

The nitric oxide-cGMP (NO-cGMP) pathway is of outstanding importance for vascular homeostasis and has multiple beneficial effects in vascular disease. Neointimal hyperplasia after vascular injury is caused by increased proliferation and migration of vascular smooth muscle cells (VSMCs). However, the role of NO-cGMP signaling in human VSMCs in this process is still not fully understood. Here, we investigate the interaction between platelet derived growth factor (PDGF)-signaling, one of the major contributors to neointimal hyperplasia, and the cGMP pathway in vascular smooth muscle, focusing on NO-sensitive soluble guanylyl cyclase (sGC). We show that PDGF reduces sGC expression by activating PI3K and Rac1, which in turn alters Notch ligand signaling. These data are corroborated by gene expression analysis in human atheromas, as well as immunohistological analysis of diseased and injured arteries. Collectively, our data identify the crosstalk between PDGF and NO/sGC signaling pathway in human VSMCs as a potential target to tackle neointimal hyperplasia.

## Abstract (web summary)

PDGF reduces expression of nitric oxide-sensitive soluble guanylyl cyclase (NO-sGC) through PI3K-P-Rex1-Rac1 signaling in vascular smooth muscle cells. These insights provide possible avenues to prevent dysregulation of NO/cGMP signaling in vascular disease.

## Introduction

Neointimal hyperplasia is an important healing process in injured vessels and a common obstacle to the long-term success of vascular interventions. Neointimal hyperplasia is characterized by excessive accumulation of VSMCs in the vascular lumen, and causes stenosis following angioplasty, stent implantation, bypass grafting, and creation of surgical anastomoses^1–3^. Additionally, neointima formation is a contributing factor to vascular occlusion in peripheral artery disease (PAD)^4^. Although VSMCs in the medial arterial layers of healthy vessels are normally quiescent and regulate vascular tone, vascular injury induces the release of growth factors and inflammatory cytokines that activate VSMCs and promote migration into the lumen of the vessel^5,6^. Once in the lumen, VSMCs undergo clonal expansion leading to vascular stenosis and ultimately ischemia^7,8^. Platelet-derived growth factor (PDGF) plays a key role in the induction of VSMC migration and proliferation^6^. PDGF is one of the most potent mitogens known, and 40–65% of post-injury vascular occlusion has been attributed to PDGF signaling^5,9,10^. Canonical PDGF signaling involves activation of mitogen-activated protein kinases (MAPKs), NF-κB, and STAT-signaling^11^, leading to VSMC de-differentiation and upregulation of genes involved in cell migration and proliferation^12,13^. Additionally, PDGF can directly regulate actin dynamics through either the Rac1-GEF Kalirin, leading to lamellipodia extension and increased migration^14^, or through the RhoA-ROCK signaling pathway^15^.

The NO-cGMP signaling cascade is a key pathway in vascular biology. NO is produced by endothelial nitric oxide synthase (eNOS) and diffuses to neighboring VSMCs, where it activates sGC, the major receptor for NO. Activation of sGC initiates the cGMP signaling cascade, which regulates vascular smooth muscle tone, VSMC plasticity, and migration^16–19^. Several studies have also suggested a role of basal NO release in preventing vascular remodeling: mice deficient in eNOS show exacerbated neointima formation after injury even in the presence of anti-hypertensive drugs^20^. Additionally, the eNOS substrate l-arginine has been shown to reduce neointima growth while the NOS inhibitor L-NAME increases it, partially independent of changes in blood pressure^21,22^. Although the overall beneficial effects of NO has prompted much research targeting components of the NO-cGMP pathway to reduce neointima formation, the results are so far not conclusive: Several animal studies have shown great promise in reducing vascular occlusion by local gene transfer of NO synthases^23^, sGC^24^, and protein kinase G (PKG, the primary receptor for cGMP)^25^. NO donors, NO-independent sGC activators, and cGMP-specific phosphodiesterase (PDE) inhibitors have also been successfully used in animal models of neointima formation^19,26–29^. Nevertheless, two human trials have investigated the effect of NO donors on restenosis after coronary balloon angioplasty^30,31^, and only one showed a modest decrease in restenosis rate, but no effect on luminal narrowing or clinical outcome^31^. This discrepancy underlines the importance of further studies into the regulation of the NO-cGMP pathway and its potential interaction with the PDGF pathway in VSMC. While cross-talk between PDGF signaling and particulate guanylate cyclases has previously been demonstrated^32,33^, no studies have so far investigated the effects of PDGF on the NO-cGMP pathway in SMCs.

Here, we present evidence of a cross-talk between the PDGF signaling pathway and the NO-cGMP pathway in human VSMCs. We show that activation of the PDGFβ receptor in VSMCs reduces the expression of sGC in neighboring human VSMCs by interfering with Notch signaling. The reduction in sGC expression renders the cells insensitive to NO-induced inhibition of migration. These data provide a possible explanation for the failure of NO donors in preventing neointima formation in human trials, and identifies potential molecular targets for intervention in cardiovascular diseases.

## Results

### PDGF-BB reduces NO-cGMP signaling in human aortic smooth muscle cells

**Fig. 1:**
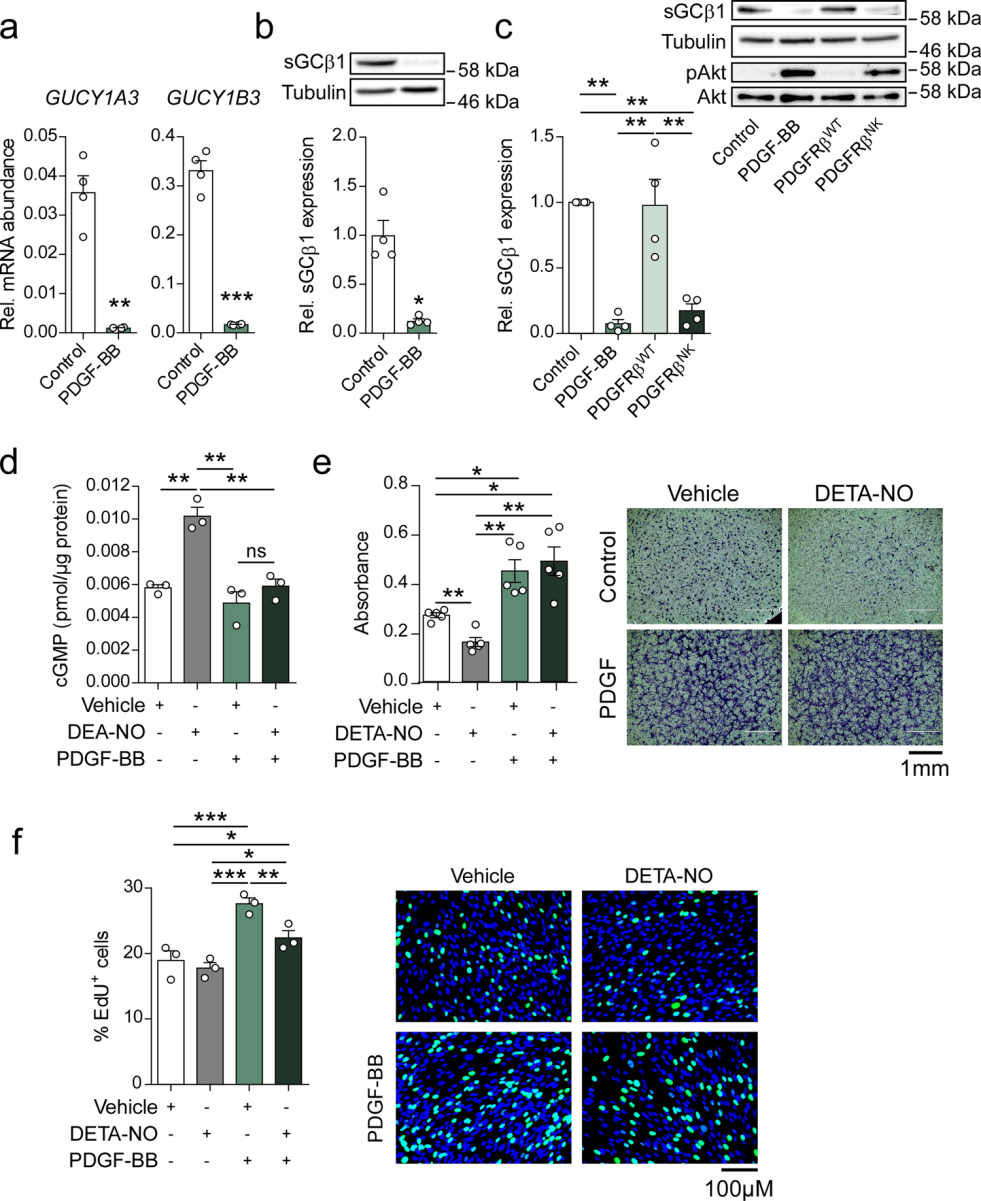
PDGF-BB reduces sGC expression and the inhibitory effects of NO on VSMC migration. **a** *GUCY1A3* and *GUCY1B3* mRNA levels in hASMCs after treatment with PDGF-BB (100 ng/ml); *t* test; *n* = 4 independent experiments. **b** sGCβ1 protein expression in hASMCs after treatment with PDGF-BB; *t* test; *n* = 4 independent experiments. **c** sGCβ1 protein expression in hASMCs after treatment with PDGF-BB or transduction with either PDGFRβ^WT^ or PDGFRβ^NK^; ANOVA with Tukey’s multiple comparisons test; *n* = 4 independent experiments. **d** NO-induced (100 µM DEA-NO) cGMP production in hASMCs after treatment with PDGF-BB; ANOVA with Tukey’s multiple comparisons test; *n* = 3 independent experiments. **e** NO-induced (100 µM DETA-NO) inhibition of hASMC migration after treatment with PDGF-BB; ANOVA with Tukey’s multiple comparisons test; *n* = 5 independent experiments. Representative images from 5 independent replicates. **f** NO-induced inhibition (100 µM DETA-NO) of hASMC proliferation after treatment with PDGF-BB; ANOVA with Tukey’s multiple comparisons test; *n* = 3 independent experiments. Representative images from 3 independent replicates. Bars indicate means ± SEM. *p < 0.05, **p < 0.01, ***p < 0.001.

To study the effect of PDGF signaling in VSMC, we stimulated human aortic VSMCs (hASMCs) with recombinant PDGF-BB. Treatment of hASMCs with PDGF significantly suppressed the mRNA levels of both the beta subunit (sGCβ)—carrying the catalytic moiety—and the regulatory alpha subunit (sGCα) of sGC (by 95% and 97%, respectively) (Fig. 1a). PDGF also significantly reduced the levels sGCβ1 protein by 87% (Fig. 1b). Expression of *PRKG1* was not significantly altered (Supplementary Fig. 1a). Transduction of hASMCs with a lentiviral vector carrying a constitutively active N666K-mutant of PDGF receptor β (PDGFRβ^NK^), which has recently been identified in patients with familial infantile myofibromatosis^34^, also significantly reduced sGCβ1 protein by 83% (Fig. 1c) indicating that PDGF-BB reduces sGC expression in hASMCs by activating the PDGFRβ. Concomitant to the reduction of sGC levels, PDGF-treatment also suppressed NO-dependent cGMP production (Fig. 1d), despite a decrease in the expression of *PDE5A* (Supplementary Fig. 1b). These data show that PDGF-signaling interferes with NO/cGMP signaling pathway via inhibition of sGC expression.

To test the functional relevance of these effects, we studied the migration of hASMCs, which has been shown to be inhibited by the NO-cGMP pathway^29,35,36^. As expected, treatment of control cells with diethylenetriamine nitric oxide adduct (DETA-NO, 100 µM) reduced migration by 40% in a Boyden chamber assay. In stark contrast, DETA-NO elicited no significant effect on migration in PDGF-treated cells (Fig. 1e). Scratch assays showed similar results, with a 44% reduction in migration after treatment with DETA-NO in control cells, but no effect in PDGF-treated cells (Supplementary Fig. 1c). Treatment with 8-Br-cGMP, on the other hand, reduced migration in both control and PDGF-treated cells (Supplementary Fig. 1d). NO-induced inhibition of proliferation, which is known to be independent of sGC/cGMP signaling^37,38^, was preserved (Fig. 1f). These results suggest that PDGF signaling prevents NO-induced inhibition of hASMC migration by reducing sGC expression.

### PDGF-BB suppresses sGC expression through PI3K and Rac1

**Fig. 2:**
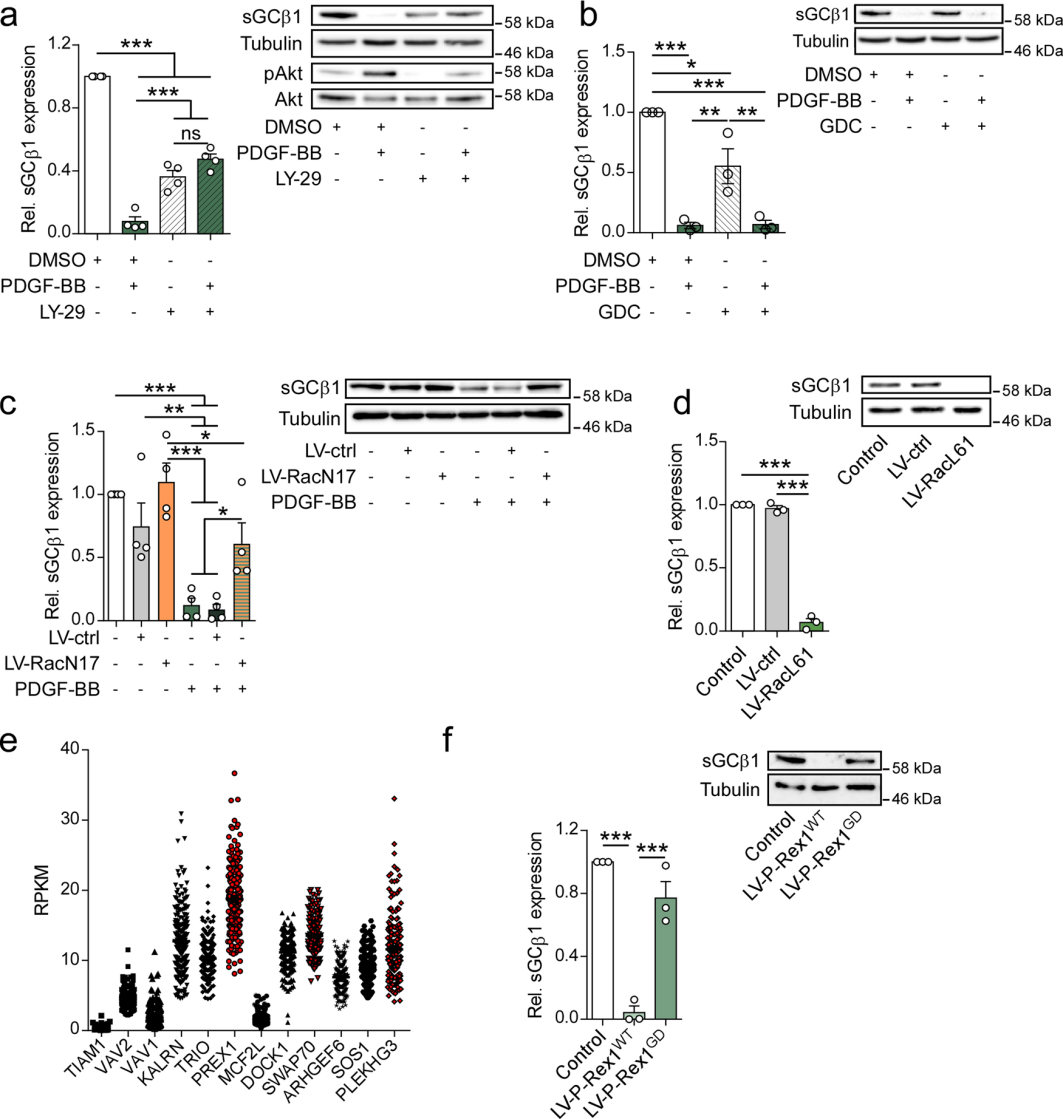
PDGF-BB reduces sGC expression via a PI3K-Rac1 signaling axis. **a** sGCβ1 protein expression in hASMCs after treatment with PDGF-BB with or without LY-294002 (LY-29, 10 µM); *n* = 4 independent experiments. **b** sGCβ1 protein expression in hASMCs after treatment with PDGF-BB with or without GDC-0068 (GDC, 1 µM); *n* = 3 independent experiments. **c** sGCβ1 protein expression in hASMCs after treatment with PDGF-BB with or without lentiviral transduction with RacN17 (LV-RacN17) or control vector (LV-ctrl); *n* = 4 independent experiments. **d** sGCβ1 protein expression in hASMCs after lentiviral transduction with RacL61 (LV-RacL61) or control vector (LV-ctrl); *n* = 3 independent experiments. **e** Expression of Rac1-activating GEFs in human aortas. Sample data obtained from the GTEx Portal (https://www.gtexportal.org/). GEFs strongly activated by PIP3 are marked in red. **f** sGCβ1 protein expression in hASMCs after lentiviral transduction with P-Rex1^WT^ (LV-P-Rex1^WT^) or P-Rex1^GD^ (LV-P-Rex1^GD^); *n* = 3 independent experiments. ANOVA with Tukey’s multiple comparisons test. Bars indicate means ± SEM. *p < 0.05, **p < 0.01, ***p < 0.001.

Next, we set out to identify the signaling pathway downstream of PDGFRβ regulating sGC expression. PDGFRβ is known to signal via different signaling cascades, including the PI3K pathway^11^. We found that treatment with the pan-PI3K inhibitor LY-294002 abolished PDGF-induced inhibition of sGC expression (Fig. 2a). Interestingly, PDGF retained its ability to downregulate sGC after inhibition of the canonical downstream target of PDGF-PI3K signaling, Akt: PDGF suppressed sGC expression also in presence of the Akt inhibitor GDC-0068 (Fig. 2b). Apart from Akt, PIP3 generated by PI3K can activate several guanine exchange factors that control activation of Rac1. We therefore transduced hASMC with a viral vector expressing dominant-negative Rac1 (RacN17) and measured PDGF-dependent sGCβ1 repression. Importantly, RacN17 expressing cells expressed significantly more sGC after treatment with PDGF-BB than cells transduced with the control virus (Fig. 2c), while expression of the constitutively active Rac1 mutant RacL61 recapitulated the effects of PDGF-treatment, and significantly reduced sGCβ1 expression by 93% (Fig. 2d). These data suggest that PDGF exerts its effect on sGC expression via Rac1 activation.

Several Rac1-activating GEFs contain PH domains and can be activated by PIP3. Transcriptome analysis of human aortas using the GTEx library revealed that *PREX1* was most highly expressed among GEFs that are sensitive to PIP3 (Fig. 2e)^39^. Indeed, overexpression of P-Rex1 significantly reduced sGCβ1 protein expression by 97% (Fig. 2f). In contrast, overexpression of the GEF-dead E56A/N238A P-Rex1 (P-Rex1^GD^) mutant did not result in any significant change in sGCβ1 protein expression (Fig. 2f), suggesting that P-Rex1 reduces sGCβ1 expression through Rac1 activation. Interestingly, CRISPR/Cas9-mediated knockdown of P-Rex1 led to a strong reduction of sGCβ1 expression, indicating that functional and balanced P-Rex1 signaling is required to maintain sGCβ1 expression in hASMCs (Supplementary Fig. 2a). It has previously been shown that the α isoform of PI3K activates Rac1 via P-Rex1 independently of Akt^40^. We therefore co-treated VSMCs with PDGF-BB and the PI3Kα-specific inhibitor Alpelisib. This led to an almost complete rescue of the PDGF-BB-mediated downregulation of *GUCY1B3* mRNA expression (Supplementary Fig. 2b). Taken together, these data point to a PDGFRβ-PI3Kα-P-Rex1-Rac1 signaling axis regulating sGC expression in hASMCs.

### The PDGF-PI3K-Rac1 pathway alters Notch signaling

To further investigate how PDGF-Rac1 signaling reduces sGC expression in hASMCs, we focused on Notch signaling, since several recent reports have demonstrated that Notch signaling regulates sGC expression in several cell types^41–43^; however, nothing is known so far about a potential link between PDGF-Rac1 signaling and the Notch pathway. Initially, we measured sGCβ1 content in hASMCs at different stages of confluence and found that sGCβ1 expression was markedly induced by cell-cell contact (Supplementary Fig. 3a). Therefore, we further investigated the Notch signaling pathway as a possible regulator of sGC expression downstream of PDGF-Rac1.

**Fig. 3:**
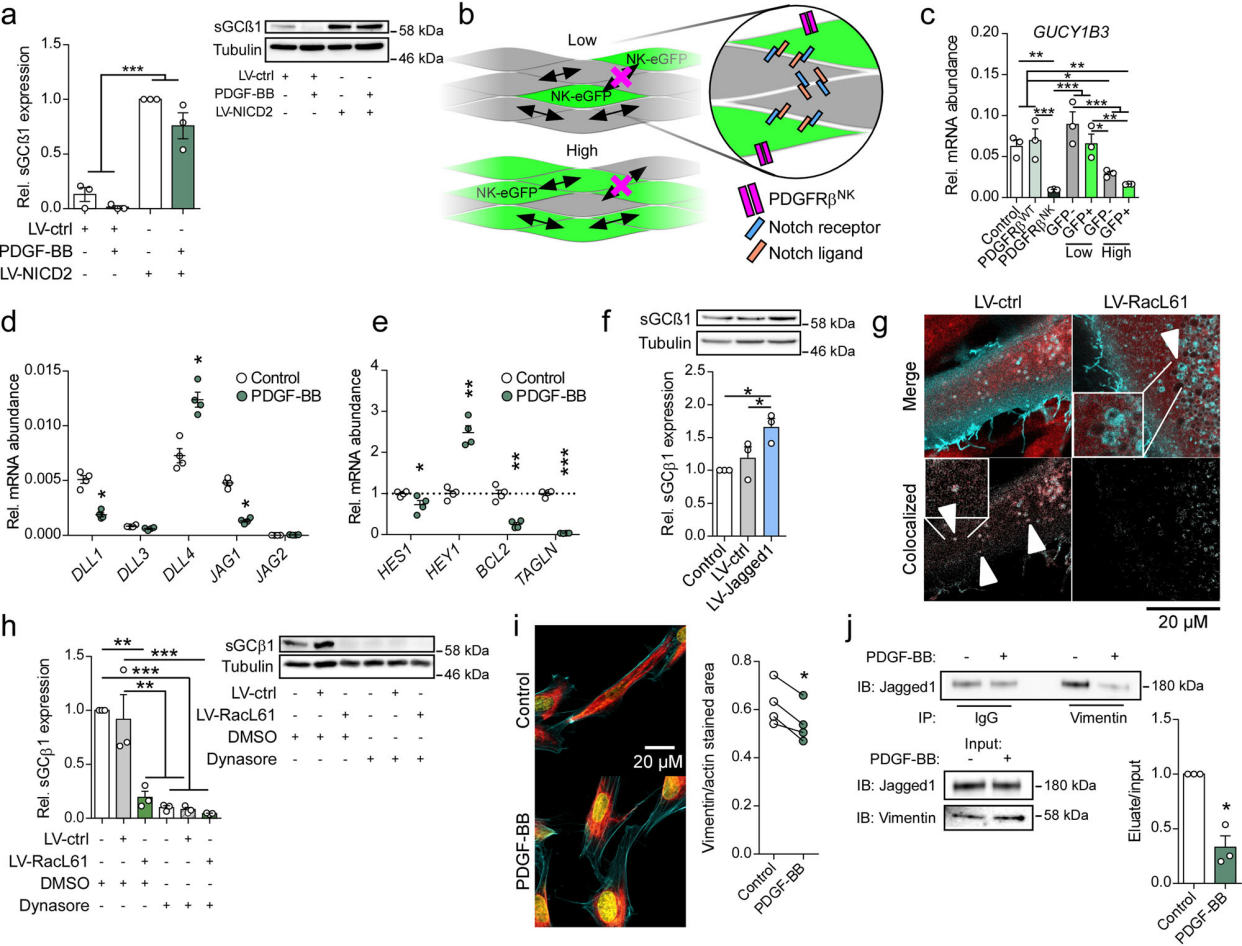
PDGF/Rac1 reduces sGC expression by interfering with Notch ligand signaling. **a** Western blot analysis of sGCß1 protein levels in N2ICD-expressing hASMCs with or without treatment PDGF-BB; ANOVA with Tukey’s multiple comparisons test; *n* = 3 independent experiments. **b** Schematic of mosaic cell culture model used to differentiate between signal-sending and signal-receiving cells (NK: PDGFRβ^NK^). **c** qPCR analysis of *GUCY1B3* expression in mosaic cell culture model of WT and PDGFRβ^WT^/PDGFRβ^NK^-expressing hVSMCs; ANOVA with Tukey’s multiple comparisons test; *n* = 3 independent experiments. **d** qPCR analysis of Notch ligand expression in hASMCs treated with PDGF-BB; Multiple *t* tests with Holm–Sidak’s correction for multiple comparisons; *n* = 4 independent experiments. **e** qPCR analysis of Notch target genes in hASMCs treated with PDGF-BB; Multiple *t* tests with Holm–Sidak’s correction for multiple comparisons; *n* = 4 independent experiments. **f** Western blot analysis of sGCß1 protein levels after lentiviral transduction with Jagged1 (LV-Jagged1) or control vector (LV-ctrl); ANOVA with Tukey’s multiple comparisons test; *n* = 3 independent experiments. **g** Colocalization of GFP-tagged Jagged1 (cyan) and mCherry-tagged Rab4a (red) after lentiviral transduction of RacL61 (LV-RacL61, right) or control vector (LV-ctrl, left). Bottom panels show colocalized pixels. Arrows indicate Jagged1+ vesicles. Representative images from 3 independent replicates. **h** Western blot analysis of sGCß1 protein levels in hASMCs after lentiviral transduction with RacL61 (LV-RacL61) or control vector (LV-ctrl) with or without concomitant treatment with Dynasore (50 µM); ANOVA with Tukey’s multiple comparisons test; *n* = 3 independent experiments. **i** Immunostaining for Vimentin (red, actin in cyan, nucleus in yellow) after treatment with PDGF-BB (left, representative images from 4 independent replicates) and calculated ratio of vimentin-to-actin stained area (right); *t* test; *n* = 4 independent experiments. **j** Co-immunoprecipitation of Jagged1 and Vimentin in hASMCs with or without treatment with PDGF-BB; *t* test; *n* = 3 independent experiments. Bars indicate means ± SEM. *p < 0.05, **p < 0.01, ***p < 0.001.

Analysis of Notch expression revealed that the hASMCs expressed Notch1, 2 and 3, with Notch2 showing the highest levels (Supplementary Fig. 3b). To mimic activated Notch signaling, we overexpressed the intracellular domain of Notch2, N2ICD. Overexpression of N2ICD led to a significant increase in mRNA expression of the canonical Notch targets *HEY1* and *HES1* (Supplementary Fig. 3c). Interestingly, hASMC expressing N2ICD did not show significantly reduced sGC expression following PDGF-treatment (Fig. 3a), indicating that PDGF modulates the NO-cGMP pathway by interfering with Notch signaling.

As Notch signaling depends on both functional Notch ligands as well as Notch receptors, we then investigated whether PDGF acts on the “signal-sending” or “signal-receiving” cell. For this, we transduced hASMCs with a lentivirus carrying a bicistronic PDGFRβ^NK^-IRES-eGFP construct at either low or high viral loads (Fig. 3b). This enabled us to coculture GFP-expressing PDGFRβ^NK^-mutant cells with wild-type (WT) cells in a mutant-dominant or WT-dominant setting. We then used fluorescence-activated cell sorting (FACS) to separate the GFP-positive (PDGFRβ^NK^ active mutant), and GFP-negative (WT) populations, and analyzed mRNA expression of *GUCY1B3* by quantitative real-time polymerase chain reaction (qRT-PCR). Interestingly, there was no significant reduction in *GUCY1B3* expression in cells expressing the mutant receptor (GFP+, low) when surrounded by wild-type (GFP−, low) cells indicating that Notch receptor signaling is functional in the cells expressing PDGFRβ^NK^ (Fig. 3c). After transduction with a high dose of PDGFRβ^NK^, GUCY1B3 expression was reduced in both cells expressing the mutant receptor (GFP+, high) as well as WT cells (GFP−, high) (Fig. 3c). This indicates reduced Notch receptor activation in wild-type cells, and altered Notch ligand function in cells expressing PDGFRβ^NK^. Coculture of GFP+ hASMCs with RacL61+GFP− cells yielded similar results, with unaltered sGC expression in RacL61+ cells surrounded by WT cells (Supplementary Fig. 3d, e). These results strongly indicate that PDGF reduces sGC expression primarily by interfering with Notch signaling in the “signal-sending” cell.

Notch signaling results in different phenotypes depending on which Notch ligands are dominant in several cell types and contexts^44–47^. Therefore, we aimed to identify which Notch ligand is involved in the PDGF-induced regulation of sGC. Analysis of Notch ligand expression pattern showed that PDGF-treatment resulted in a significant, 4.4-fold increase in expression of Dll4 and a significant decrease in expression of Jagged1 and Dll1 by 72% and 63%, respectively (Fig. 3d). Since activation of Notch signaling in VSMCs by endothelial Jagged1 is known to promote VSMC differentiation^48^, and Jagged1 and Dll4 have inverse effects on angiogenesis^44^, we focused on these two Notch ligands. Dll4 and Jagged1 participate in positive feedback mechanisms resulting in bistable signaling circuits, with each ligand regulating its own expression^49,50^. We therefore wondered if PDGF signaling could alter Notch ligand signaling. Ligand-specific differences in Notch signaling are not well understood and are complicated by e.g. *cis*-inhibition (i.e. inhibition of Notch by ligands on the same cell)^44^. Nevertheless, several Notch target genes are differentially regulated by Jagged1 and Dll4 in human vascular tissue, including *BCL2*, *TAGLN, HEYL*, and *HEY2*^47^. PDGF-treatment strongly reduced the mRNA expression of *BCL2* and *TAGLN* by 75% and 97%, respectively (Fig. 3e), suggesting reduced Jagged1 activity. Additionally, Hey1 expression was recently shown to be preferentially regulated by Dll4^46^. Accordingly, PDGF-treatment significantly increased Hey1 expression by 248%, suggesting an increase in Dll4 activity (Fig. 3e). The canonical Notch target gene *HES1* showed modest reduction (27%) in expression and has previously been associated with both Jagged1 and Dll4 signaling^51,52^. These data, together with increased Dll4 expression and reduced Jagged1 expression, indicate that PDGF-treatment of hASMCs induces a Notch target gene expression profile consistent with a switch in Notch ligand activity from Jagged1 to Dll4. Importantly, lentiviral overexpression of Jagged1 significantly increased sGCβ1 expression by 66%, suggesting a specific role for Jagged1 in maintaining sGCβ1 expression in hASMCs (Fig. 3f). Next, we focused on identifying the link between Rac1 and Notch ligand signaling. Notch ligand endocytosis is critical in initiating Notch signaling, either by ligand activation via endosomal recycling, or by exerting a “pulling force” allowing for cleavage and activation of Notch^53^. In most cells, clathrin-mediated endocytosis and endosomal recycling of Notch ligands are considered to be a crucial step in Notch signaling^54^. Furthermore, Rac1 is an important regulator of both CME and endosome recycling^55–58^. We therefore studied the effect of Rac1 activity on endocytotic processing of Jagged1 by analyzing colocalization of mCherry-tagged Rab4a, a marker for recycling endosomes^59^, with GFP-tagged Jagged1 in hASMCs transduced with either RacL61 or an empty control vector. In control cells, Rab4a was found colocalized with Jagged1 in cytosolic vesicles, indicating endosomal recycling of Jagged1 and functional Jagged1 signaling (Fig. 3g). In contrast, Jagged1-positive vesicles were mostly found accumulated in the perinuclear region of cells expressing RacL61 (Fig. 3g). These vesicles were negative for Rab4a. Additionally, blocking endocytosis with the dynamin inhibitor Dynasore reduced sGC expression to a similar extent as overexpressing RacL61 (Fig. 3h). These data suggest that functional endocytotic pathways are required to maintain sGC expression, and that activating Rac1 interferes with Jagged1 endocytotic processing.

Jagged1-Vimentin interaction is necessary for full Jagged1 signaling^15^, and the Vimentin cytoskeleton is implicated in Rab4+ endosomal trafficking^60^. Additionally, Rac1 and PDGFRB activation can reorganize the Vimentin cytoskeleton in fibroblasts and endothelial cells^61,62^, the latter via a PI3K-dependent mechanism^61^. Immunostaining of Vimentin showed that treatment with PDGF for 30 min in hASMCs led to a significant retraction of Vimentin from the cell membrane (Fig. 3i). Furthermore, co-immunoprecipitation of Vimentin in Jagged1-overexpressing hASMCs revealed that PDGF strongly reduces the ability of Jagged1 to bind to Vimentin (Fig. 3j). The activation of Jagged1 through its interaction with Vimentin has been shown to be dependent on the phosphorylation of the N-terminal domain of Vimentin^15^. Interestingly, ectopic overexpression of a phosphodeficient S4,6,7,8,9A-Vimentin mutant strongly reduced sGC expression (Supplementary Fig. 3f), demonstrating that a functional dynamic Vimentin cytoskeleton is required for maintained sGC expression in hASMCs. These data suggest a mechanistic link between Rac1 activation and dysregulation of the endocytosis step of Jagged1 activation, possibly through rearrangement of the Vimentin intermediate filaments.

### sGC expression is differentially regulated in murine and human VSMCs

To test whether this crosstalk between PDGF and sGC/cGMP is also functional in other species, we focused on the mouse because of the potential use of genetic models. PDGF-BB also reduced sGCβ1 expression in murine VSMCs, albeit to lower degree than in human cells (Supplementary Fig. 4a). Surprisingly, RacL61 expression induced both sGCβ1 expression (Supplementary Fig. 4b) and phosphorylation of the PKG target RhoA (Supplementary Fig. 4c), indicating increased cGMP signaling. The discrepancy between the responses of PDGFR and Rac1 activation in human and murine VSMCs suggests that there are considerable interspecies differences in the transcriptional regulation of sGC. Indeed, Needleman–Wunsch alignment of the human *GUCY1A3* and *GUCY1B3* promoters with those of common laboratory animals showed very low NW scores and identities (Supplementary Data 1). These data indicate that sGC expression is differentially regulated by PDGF and Rac1 in human and murine VSMCs.

### PDGF correlates with altered Notch signaling and reduced sGC expression in atheromas

**Fig. 4:**
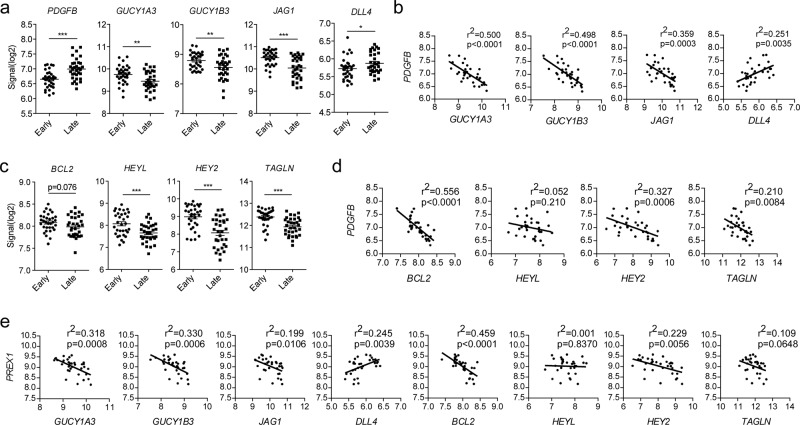
PDGFB and PREX1 correlates with Notch ligand switching and negative sGC regulation in human carotid artery atherosclerotic plaques. **a** mRNA expression of *PDGFB*, *GUCY1A3*, *GUCY1B3*, *JAG1*, and *DLL4* in early and late stage atherosclerotic lesions. **b** Correlation of *PDGFB* with *GUCY1A3*, *GUCY1B3*, *JAG1*, and *DLL4* expression in late stage atheromas. **c** mRNA expression of Notch target genes *BCL2*, *HEYL*, *HEY2*, and *TAGLN* in early and late stage atherosclerotic lesions. **d** Correlation of *PDGFB* with *BCL2*, *HEYL*, *HEY2*, and *TAGLN* expression in late stage atheromas. **e** Correlation of *PREX1* with *GUCY1A3*, *GUCY1B3*, *JAG1*, *DLL4*, *BCL2*, *HEYL*, *HEY2*, and *TAGLN* expression in late stage atheromas. Sample data obtained from GEO dataset GSE43292. Correlation analysis was performed with Pearson’s correlation test.

Next, we focused on the expression of sGC in human vascular disease states that are characterized by active PDGF signaling. First, we analyzed mRNA expression in carotid arteries with early stage atheroma (stage I-II) versus late stage (stage IV and higher, data taken from GEO dataset GSE43292). As expected, PDGFB expression was significantly increased in the late-stage atheroma samples compared to early stage (Fig. 4a). Importantly, *GUCY1A3*, *GUCY1B3*, and *JAG1* were significantly decreased in the late-stage plaques, whereas DLL4 was increased (Fig. 4a). In parallel, *GUCY1A3*, *GUCY1B3*, and *JAG1* all showed strong negative correlation with *PDGFB* in late-stage samples (Fig. 4b). Conversely, DLL4 correlated positively with *PDGFB* (Fig. 4b). This suggests that pathological activation of VSMCs by PDGF in vivo promotes Notch ligand switching. Corroborating this notion, downstream targets of Jagged1 signaling, *BCL2*, *HEYL*, *HEY2*, and *TAGLN* were all decreased in late-stage atheroma samples (Fig. 4c). *PDGFB* expression in late-stage atheroma samples also negatively correlated with expression of *BCL2*, *HEY2*, and *TAGLN* (Fig. 4d). These expression patterns were also observed in early stage lesions, but to a lesser extent (Supplementary Fig. 5a, b). These data strongly support our in vitro findings and demonstrate that PDGF signaling correlates with Notch ligand switching and decreased sGC expression in human atheromas. As our in vitro experiments pointed to P-Rex1 as the GEF responsible for PDGF-induced Rac1 activation and subsequent interference with Notch signaling, we also performed correlation analysis for *PREX1* in the same samples. We observed significant negative correlation between *PREX1* and *GUCY1A3*, *GUCY1B3*, and *JAG1*, and positive correlation between *PREX1* and *DLL4* in late-stage atheromas (Fig. 4e). Furthermore, we found negative correlation between *PREX1* and the *JAG1*-responsive genes *BCL2*, *HEY2*, and *TAGLN* in late stage samples (Fig. 4e). *GUCY1B3*, *BCL2*, and *HEY2* also correlated negatively with *PREX1* in early stage samples (Supplementary Fig. 5c, d). Again, these data strongly argue for a crosstalk between PDGF- and cGMP signaling via Notch ligands in vascular remodeling. We validated these data with a second cohort of samples collected from early stage lesions, which showed negative correlation between the expression of *PDGFB* and *GUCY1B3*, *JAG1*, *HEYL*, and *HEY2* (Supplementary Fig. 6a, b).

### sGC expression is reduced in human neointimal tissue

**Fig. 5:**
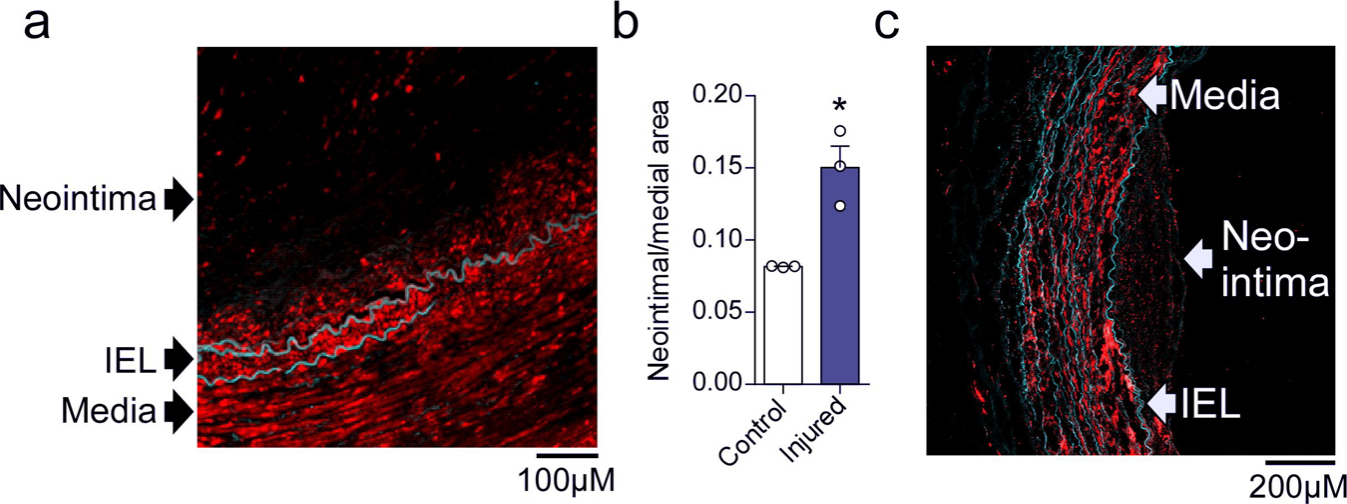
sGCβ1 expression is reduced in the neointima of diseased and injured human arteries. **a** Immunostaining showing sGCβ1 protein expression (red color) in diseased arteries from PAD patients. IEL: Internal elastic lamina. **b** Neointima-to-media ratio in control and injured IMA segments; *t* test; *n* = 3 independent experiments. **c** Immunostaining showing sGCβ1 protein expression in an injured IMA segment. IEL: Internal elastic lamina. Cyan color is autofluorescence from the elastic lamina. Bars indicate means ± SEM. Representative images from 3 biological replicates. *p < 0.05.

To directly test our hypothesis, we focused on peripheral artery disease (PAD), which is characterized by a pronounced neointimal hyperplasia and a dramatic increase in *PDGF-B* expression in the vascular wall^63^. We hypothesized that the neointima, containing migrated PDGF-activated VSMCs, should have decreased sGC expression. Therefore, we analyzed the expression of sGC in the media and neointima of arteries from patients with PAD. Immunostaining for sGCβ1 showed high expression in the medial layer but only faint signal in the neointima (Fig. 5a). Additionally, in situ hybridization using RNAscope revealed a higher density of *GUCY1B3* transcripts in the media compared to the neointima (Supplementary Fig. 7). Next, we examined sGCβ1 expression in human internal mammary arteries (IMAs) subjected to ex vivo balloon angioplasty, a well-established model that recapitulates early stages of injury-induced intimal hyperplasia^64–66^. The injured vessels developed neointimal hyperplasia after two weeks in culture (Fig. 5b), and immunohistochemical analysis revealed a much lower staining intensity of sGCβ1 in the neointima compared to the media (Fig. 5c). These results suggest that human neointimal VSMCs have low expression of sGC and are therefore unlikely to be sensitive to NO-releasing drugs.

## Discussion

Abnormal accumulation of VSMCs in the lumen of blood vessels is a major detrimental factor in vascular pathologies such as PAD, vein graft stenosis, in-stent restenosis, post-angioplasty restenosis, and stenosis of arteriovenous fistulae^1–4^. Despite initial conflicting reports^67^, there is now strong evidence that, at the initiation of neointima formation, a small number of activated SMCs migrate to the vascular lumen where they undergo clonal expansion, eventually leading to occlusion of the vessel^7,8,67^. Dysregulated SMC migration is therefore a key process in the early establishment of neointimal hyperplasia.

In the present study, we investigated the PDGF-induced effects in VSMC focusing on NO/cGMP signaling pathway. Our data reveal a cross-talk between these two major signaling pathways involved in VSMC migration: PDGF- and cGMP signaling. These signaling cascades play opposing roles in neointima formation^9,10,20–22,24–29^. We found that PDGF-BB suppresses the expression of sGC through PDGFRβ, and therefore disables NO-mediated cGMP signaling in VSMCs. Consequently, NO-releasing drugs were ineffective in reducing VSMC migration following treatment with PDGF-BB.

Downstream of the PDGF receptor, we identified the PI3K-P-Rex1-Rac1 signaling axis as mediator of this effect. Curiously, we saw the same defect in sGC expression upon both overexpressing P-Rex1 and with CRISPR-Cas9-based knockdown of P-Rex1. P-Rex1 is a multi-domain protein with both GEF and scaffolding properties, and is known to regulate endocytosis in Rac1-dependent manner^68^. Intriguingly, both scaffold proteins as well as proteins involved in endocytotic machinery have been known to generate the same phenotypes when either overexpressed or silenced^69,70^. In particular, silencing or over-expressing intersectins, which also are multi-domain scaffold proteins acting as Rho-GEFs, generates the same defects in endocytosis^69^.

Interestingly, P-Rex1/Rac1 is known to cooperate with PDGFRβ in cancer cell invasion, which, along with neointimal hyperplasia, are classical examples of dysregulated cellular migration^71^. It is important to also note that metastatic cancer cells have been reported to lack sGCβ1, and are therefore also resistant to NO-induced cGMP generation^72^. Our data clearly suggest that the PDGFRβ-P-Rex1-Rac1 pathway is also highly relevant in pathological VSMC migration. Investigation into a potential role for PDGF in the regulation of NO-cGMP signaling in cancer cells would be warranted.

Transcriptional control of the sGC subunits is not fully understood. Analysis of the promoter regions of human *GUCY1A3* and *GUCY1B3* has revealed putative binding sites for, amongst others, NFκB, SP1, NFY, and CCAAT-binding factors^19^. In recent years, an important role of Notch signaling in the transcriptional regulation of sGC has also emerged^41–43^. The sGC promoter harbors several binding sites for the Notch-dependent transcription factor recombining binding protein suppressor of hairless κ (RBPJκ), and is thus regulated by Notch signaling in several different cell types including VSMCs^41–43^. In the present study, we showed that the ability of PDGF-BB to reduce sGC expression in human VSMCs was blunted by overexpressing the intracellular domain of Notch2, strongly suggesting a role for PDGF in the regulation sGC expression via interference with Notch signaling. Notch signaling can give rise to distinct cellular phenotypes depending on specific ligand-receptor pairing. By analyzing the expression of Notch target genes, we found that PDGF appears to bring about a switch in Notch ligand activity: in the absence of PDGF, Jagged1 signaling is predominant, while activation of PDGF signaling leads to a gene expression pattern consistent with increased Dll4 signaling and reduced Jagged1 signaling. Our data suggest that the loss of Jagged1 signaling caused by PDGF-treatment abrogates sGC expression in hASMCs. These data are in line with previous studies showing that Jagged1 signaling increases sGC expression in VSMCs^43^.

Previous studies have shown that atherosclerotic rabbit arteries have reduced sGC expression^73^, and that balloon- and wire injury of rat arteries leads to a transient reduction of sGC expression in the vascular media^19,24^. However, at least one study in rats showed that the sGC expression is elevated in neointimal cells post-injury^19^. This stands in contrast to our results which clearly show strongly reduced sGC expression in the neointima of arteries from PAD patients, as well as in the neointima of *ex viv*o injured human IMAs. We found that while treatment with PDGF reduces sGC expression in murine VSMCs in vitro, this effect is much less prominent and possibly mediated via a different pathway than in human VSMCs. These data are in good agreement with previous studies, which have shown that deletion of Rac1 has no effect on sGC expression in mice^74^. These data indicate that there are important species differences regarding the PDGF-sGC crosstalk.

The apparent differential regulation in humans and laboratory animals severely limits the experimental approaches to investigate sGC expression in pathologically activated VSMCs. Additionally, there are currently no models that fully recapitulate human neointima formation in vivo. To study PDGF-mediated regulation of sGC expression in neointima formation in human vessels, we looked at several models and disease states that involve PDGF-mediated activation of VSMCs. Analyzing available datasets on mRNA expression in atheromas, we found striking correlation between or observed PDGF-induced in vitro phenotype and gene expression patterns in vivo. Importantly, we found that sGC expression is reduced in the neointima of both arteries with PAD and in an ex vivo-injury IMA model. This interspecies discrepancy may also help to explain why NO-releasing drugs have shown so poor effects on ameliorating vascular stenosis in human trials despite promising animal studies.

VSMCs play a central role in atherosclerosis: migration of VSMCs from the media to the intima is a crucial step in the progression of atherosclerosis, although its role in the pathogenesis is complex and poorly understood. It is well established that in atherosclerosis, PDGF signaling in the vessel wall is increased and that this is a major driving factor for activation and migration of VSMCs^75^. These migrating VSMCs produce pro-inflammatory agents that may accelerate the disease progression^75^. In the later stages, however, migrating VSMCs stabilize the fibrous cap and are likely protective against plaque rupture and thrombosis^75^. Thus, a better understanding of the regulation of VSMC migration and the pathways involved is of great interest for the prevention and treatment of the disease. The results presented in the present study could therefore also be of relevance to therapeutic modulation of VSMC migration in atherosclerosis. Our data provide several mechanistic points of intervention for translational studies in order to prevent dysregulation of sGC expression in activated VSMCs in vascular disease, both when beneficial (atherosclerosis) or detrimental (neointimal hyperplasia).

## Methods

### Patient samples

Patients undergoing coronary bypass surgery at the Klinik und Poliklinik für Herzchirurgie, University Clinic Bonn, Bonn, Germany, were enrolled in the study. Informed consent was obtained. All experiments using patient IMA samples were approved by the Ethics Commission at the University Clinic Bonn, Bonn, Germany (Reference 185/19). Patient PAD samples were provided by the Munich Vascular Biobank. The human gene expression datasets used in the study are available from Gene Expression Omnibus (https://www.ncbi.nlm.nih.gov/gds) with the permanent accession number GSE43292^76^, and from the GTEx portal (https://www.gtexportal.org/).

### Mice

The mice used for murine VSMC isolation were euthanized in accordance with the German Animal Welfare Act (TierSchG). No experiments on live animals were performed in this study.

### Cell culture

Primary human aortic smooth muscle cells were purchased from Provitro AG (Berlin, Germany). Cells were cultivated in smooth muscle cell growth medium (Provitro) until three days post confluence, unless indicated otherwise. For mouse VSMCs, thoracic aortas from 4 weeks old male C57Bl/6-J mice were isolated, and the perivascular fat pads and connective tissues were carefully removed. The aortas were digested with collagenase and elastase-containing isolation buffer at 37 °C for 10 min. Next, the adventitia was carefully peeled off from the rest of the aortas, after which they were cut into ~1 mm pieces and placed back in the enzymatic digestion buffer at 37 °C for 30 min. The resulting suspension was centrifuged at 500 *g* for 5 min, and the digestion buffer removed. The pellet was resuspended in SMC growth medium (Provitro), and plated in 6-well plates (cells from one aorta per well).

### Antibodies

A list of antibodies used in this study is provided in Table 1.

**Table 1:** List of antibodies used in this study.

Antigene	Manufacturer	Cat. No.	Dilution
Akt	Cell Signaling, Danvers, USA	9272S	1:1000
GAPDH	Cell Signaling, Danvers, USA	2118S	1:1000
Goat anti-Rabbit IgG AF-555	ThermoFischer Scientific, Darmstadt	A-21428	1:500
IgG isotype control	Cell Signaling, Danvers, USA	3900S	0.1 µg (IP)
Jagged-1	Cell Signaling, Danvers, USA	70109S	1:1000
Anti-Mouse-HRP	Cell Signaling, Danvers, USA	7076S	1:10000
pAkt Serine 473	Cell Signaling, Danvers, USA	9271S	1:1000
pRhoA Serine 188	Santa Cruz, Santa Cruz, USA	Sc-32954	1:1000
Anti-Rabbit-HRP	Cell Signaling, Danvers, USA	7074S	1:5000
RhoA	Santa Cruz, Santa Cruz, USA	Sc-418	1:1000
sGCβ1	Sigma-Aldrich, Munich, Germany	G4405	1:1000 (WB) 1:100 (Immunostaining)
Tubulin	Dianova, Hamburg, Germany	DLN-09993	1:1000
Vimentin	Cell Signaling, Danvers, USA	5741S	1:1000 (WB) 1:100 (Immunostaining) 0.1 µg (IP)
P-Rex1	Cell Signaling, Danvers, USA	13168S	1:1000 (WB)

### Lentiviral constructs

All constructs except LV-P-Rex1^WT^ and LV-P-Rex1^GD^ were cloned by amplifying cDNA and ligating into the vector 156rrlsinPPTCMV (provided by Luigi Naldini). P-Rex1 cDNA was kindly provided by Heidi Welch and cloned into the 156rrlsinPPTCMV vector. Point mutations in LV-PDGFRB^NK^, LV-P-Rex1^GD^, and LV-VimSA were inserted by site-directed mutagenesis by PCR with Q5^®^ Hot start High-Fidelity DNA Polymerase (New England Biolabs, Ipswitch, USA). CRISPR-Cas9 experiments were performed using the Lenticrispr V2 plasmid system. Lentiviral doses corresponding to 10–50 ng of viral reverse transcriptase per well were used for each experiment.

### qRT-PCR

cDNA was prepared using ProtoScript^®^ II First Strand cDNA Synthesis Kit (New England Biolabs, Ipswitch, USA). Quantitative real-time PCR (qPCR) was performed using SYBR™ Green PCR Master Mix (Thermo Fisher Scientific, Waltham, USA) on the Applied Biosystems^®^ StepOnePlus™ System using the primers listed in Table 2.

**Table 2:** List of qPCR primers used in this study.

BCL2_f	5′-ATG GGA TCG TTG CCT TAT GC-3′
BCL2_r	5′-AGT CTA CTT CCT CTG TGA TGT TGT-3’
DLL1_f	5′-AAG CGT GAC ACC AAG TGC C-3’
DLL1_r	5′-CTT TCA GAT GCT TCT CCA CCC C-3’
DLL3_f	5′-TCC CGG ATG CAC TCA ACA AC-3’
DLL3_r	5′-AGG GCG ATT CCA ATC TAC GG-3’
DLL4_f	5′-AGG GAC TCC ATG TAC CAG TC-3’
DLL4_r	5′-CTC CTG CCT TAT ACC TCC GT-3’
GAPDH_f	5′-ACC ATC TTC CAG GAG CGA GAC-3’
GAPDH_r	5′-GCC TTC TCC ATG GTG GTG AA-3’
GUCY1A3_f	5′-CGG AAA ATC AAT GTC AGC CC-3’
GUCY1A3_r	5′-AGG GAA GTT TGG TGG AAG CTC-3’
GUCY1B3_f	5′ -CCT TCT TCA TCT AAC TGT GCC TC-3’
GUCY1B3_r	5′-TAC GGA TTT GTG AAT CAC GC-3’
HES1_f	5′-CCC AAC GCA GTG TCA CCT TC-3’
HES1_r	5′-TAC AAA GGC GCA ATC CAA TAT G-3’
HEY1_f	5′-ACG AGA ATG GAA ACT TGA GTT C-3’
HEY1_r	5′-AAC TCC GAT AGT CCA TAG CAA G-3’
JAG1_f	5′-ATG CGT TCC CCA CGG AC-3’
JAG1_r	5′-CCC CAC ACA CCT TGG CTC-3’
JAG2_f	5′-TGC AAA AAC CTG ATT GGC GG-3’
JAG2_r	5′-CGA CAG TCG TTG ACG TTG AT-3’
NOTCH1_f	5′-CGA TGC TCC CAG CCC G-3’
NOTCH1_r	5′-CCG CCA CAG ACG CAG G-3’
NOTCH2_f	5′-CCT GTT CCC CAA ACC CTT GT-3’
NOTCH2_r	5′-ATG GTA CAC CGC TGA CCT TG-3’
NOTCH3_f	5′-CCA ACC TGG CAG GGA GTT TC-3’
NOTCH3_r	5′-TTC AGG CAT GGG TTG GGG TC-3’
NOTCH4_f	5′-GGA GAA GGG GCT GTG GAA TG-3’
NOTCH4_r	5′-CAG CAG CCC TCT GGG TCT-3’
TAGLN_f	5′-AAT TGA TGG AAA CCA CCG GG-3’
TAGLN_r	5′-GGG GAA AGC TCC TTG GAA GT-3’

### Western Blot

Cells were lysed with radioimmunoprecipitation assay (RIPA) buffer containing protease and phosphatase inhibitors. SDS-PAGE was performed in SDS-containing electrophoresis buffer at 100 V at room temperature with the Mini Trans-Blot^®^ Cell system (Bio-Rad Laboratories, Hercules, USA). After electrophoresis, the gel was removed and placed in the transfer assembly of the Mini Trans-Blot^®^ Cell system (Bio-Rad Laboratories, Hercules, USA). A PVDF membrane was activated in methanol for 15 s, equilibrated in transfer buffer, and placed on top of the gel. The proteins were transferred by application of a transverse electric field (300 mA for 90 min). After blotting, the membrane was blocked TBS with 0.1% Tween-20 (TBST) and 5% milk protein (AppliChem GmbH, Darmstadt, Germany) for 1 h. Membranes were incubated with primary antibody at 4 °C overnight, washed, and incubated with secondary HRP-conjugated antibody for 1 h at room temperature. The membrane was then washed and covered in ECL reagent for 2 min according to the manufacturer’s instructions. Luminescence was measured immediately in an ImageQuant LAS 4000 chemiluminescence reader. Densitometric analysis was performed in ImageJ.

### cGMP measurements

cGMP levels were measured using a monoclonal anti-cGMP enzymatic immunoassay (EIA) (80103, NewEast Biosciences, King of Prussia, USA) according to the manufacturer’s instructions. The protein concentration in each sample was analyzed with the Pierce™ bicinchoninic acid (BCA) Protein Assay Kit (23225, ThermoFischer Scientific) according to the supplier’s manual. The cGMP content in each sample was normalized to the protein concentration measured in the BCA assay. cGMP content was measured in the presence of 500 µM IBMX. Lysates were harvested in ice-cold 100 µM HCl.

### Migration assay

For Boyden chamber assays, QCM Chemotaxis Cell Migration Assay (Sigma-Aldrich) was used according to the manufacturer’s instructions. Scratch assays were conducted in 6-well plates. Scratches were inflicted in each well using a micropipette tip, and cells were washed once before adding compounds. At least three images per scratch were used for analysis using TScratch (https://www.cse-lab.ethz.ch/software/). In all migration experiments, cells were starved for 48 h prior to assay start in order to prevent proliferation

### Proliferation assay

Proliferation assays were performed with Click-iT™ EdU Cell Proliferation Kit for Imaging (Thermo Fischer Scientific) according to the manufacturer’s instructions. Fluorescence microscopy was performed with a Leica DMI4000 B microscope (Leica Mikrosysteme Vertrieb GmbH) equipped with a Leica DFC425 C camera (Leica Mikrosysteme Vertrieb GmbH).

### Fluorescence-activated cell sorting

FACS experiments were carried out using a BD FACSAria Fusion flow cytometer (BD Biosciences, San Jose, USA) in the Flow Cytometry Core Facility at the Institute of Experimental Immunology, Medical Faculty at the University of Bonn.

### Internal mammary artery injury model

A percutaneous transluminal coronary angioplasty catheter (2 mm Emerge™ Monorail™, Boston Scientific) was inserted into the IMA segment lumen and inflated to 15 bar. The inflated catheter was then withdrawn with simultaneous rotation. This was repeated three times, and the segment were then incubated in smooth muscle cell growth medium (Provitro) for 2 weeks at 37 °C and 5% CO_2_.

### Cryosectioning

IMA segments were fixed for 1 h with 4% PFA in PBS at 4 °C, and then placed in 20% sucrose in PBS overnight at 4 °C. The segments were then placed in a solution containing a 50/50 mix of 20% sucrose and O.C.T.™ Compound, and slowly agitated for 1 h to prevent the collapse of the vessels upon embedding. The arteries were then placed in molds, fully covered in O.C.T.™ Compound, and placed at −80 °C. Sectioning of the arteries was performed on a cryotome (CM1850, Leica) with 5 µm section thickness. The sections were transferred to Superfrost Plus microscope slides, allowed to dry for 30 min, and stored at −80 °C.

### Immunostaining

Frozen sections were allowed to thaw briefly, washed 3 × 5 min in PBS with 0,1% Tween-20 (PBST), and then incubated with blocking buffer (5% BSA in PBS) for 60 min at room temperature. The blocking buffer was then removed, and the primary antibody solution added. After incubation overnight in a humid chamber at 4 °C, sections were washed 3 × 5 min in PBST, after which the secondary antibody solution was added. The sections were incubated in darkness for 2 h at room temperature, washed 2 × 5 min, and mounted with Immu-Mount (ThermoFischer Scientific) under a coverslip.

### Fluorescence microscopy

Fluorescence microscopy was performed with a Zeiss LSM700 microscope (Carl Zeiss AG) equipped with a Zeiss Axiocam 506 color camera (Carl Zeiss AG).

### Co-immunoprecipitation

Cell transduced with LV-Jagged1 were harvested with Co-IP lysis buffer (150 mM NaCl, 25 mM Tris-HCl pH 7.4, 1% Triton-X 100, 0.5% CHAPS, 0.5% NaDOC, Complete EDTA-free protease inhibitor) for 30 min at 4 °C with constant agitation. Lysates were centrifuged and pre-cleared using Pierce™ Protein G Agarose (ThermoFischer Scientific), and incubated with anti-Vimentin or IgG isotype control antibody (Cell Signaling, ~2 mg protein per µg antibody) for 2 h at 4 °C. Protein G bead slurry was added to the samples and incubated for a further 2 h at 4 °C with constant agitation. Samples were then washed three times in Co-IP wash buffer (150 mM NaCl, 10 mM Tris-HCl pH 7.4, 1% Triton-X 100, 1 mM EDTA pH 8, Complete EDTA-free protease inhibitor), followed by elution and SDS-PAGE. All handling of cell lysates was performed on ice or in a 4 °C cold room.

### RNAscope in situ hybridization

RNAscope in situ hybridization was performed on PAD sections with the probe Hs-GUCY1B3 (Cat No. 425841, Bio-Techne) according to the manufacturer’s instructions.

### Statistics and reproducibility

All bars represent means ± SEM. In comparisons between two conditions, significance was determined with two-tailed Student’s *t* test. Where appropriate, correction for multiple comparisons was performed with the Holm–Sidak method. For comparisons between more than two conditions, statistical significance was determined by ANOVA and Tukey’s multiple comparisons test. Correlation analysis was performed with Pearson’s correlation test. Replicates are defined as independently repeated experiments.

### Reporting summary

Further information on research design is available in the Nature Research Reporting Summary linked to this article.

## Supplementary information

Supplementary Information

Description of Additional Supplementary Files

Supplementary Data 1

Reporting Summary

## Data Availability

The data that support the findings of this study are available from the corresponding author upon reasonable request. The original, uncropped blot images can be found in Supplementary Fig. 8. Source data behind the graphs can be found in the Supplementary Data.
