# Author Correction: PDGF regulates guanylate cyclase expression and cGMP signaling in vascular smooth muscle

**DOI:** 10.1038/s42003-022-03244-9

**Published:** 2022-03-28

**Authors:** Staffan Hildebrand, Mohamed Ibrahim, Andreas Schlitzer, Lars Maegdefessel, Wilhelm Röll, Alexander Pfeifer

**Affiliations:** 1 Institute of Pharmacology and Toxicology, University Hospital, University of Bonn, Bonn, Germany; 2 Quantitative Systems Biology, LIMES-Institute (Life and Medical Sciences Bonn), University of Bonn, Bonn, Germany; 3 Experimental Vascular Surgery and Medicine, Department of Vascular and Endovascular Surgery, Klinikum rechts der Isar - Technical University Munich, Munich, Germany; 4 Department of Medicine, Karolinska Institutet, Stockholm, Sweden; 5 Department of Cardiac Surgery, University of Bonn, Bonn, Germany

**Keywords:** Restenosis, Growth factor signalling

Correction to: *Communications Biology* 10.1038/s42003-022-03140-2, published online 03 March 2022.

In the original version of the Article, the order of references was incorrect in the References paragraph. The references now appear in the correct order of referencing.

This has now been corrected in the PDF and HTML versions of the Article.

