# Supplementary material for: PDGF regulates guanylate cyclase expression and cGMP signaling in vascular smooth muscle
Source: Commun Biol. 2022 Mar 3;5:197. doi: 10.1038/s42003-022-03140-2 (PMC8894477; doi:10.1038/s42003-022-03140-2)
Supplement: Supplementary file 1 — Supplementary Information [file 42003_2022_3140_MOESM1_ESM.pdf]

SUPPLEMENTARY INFORMATION

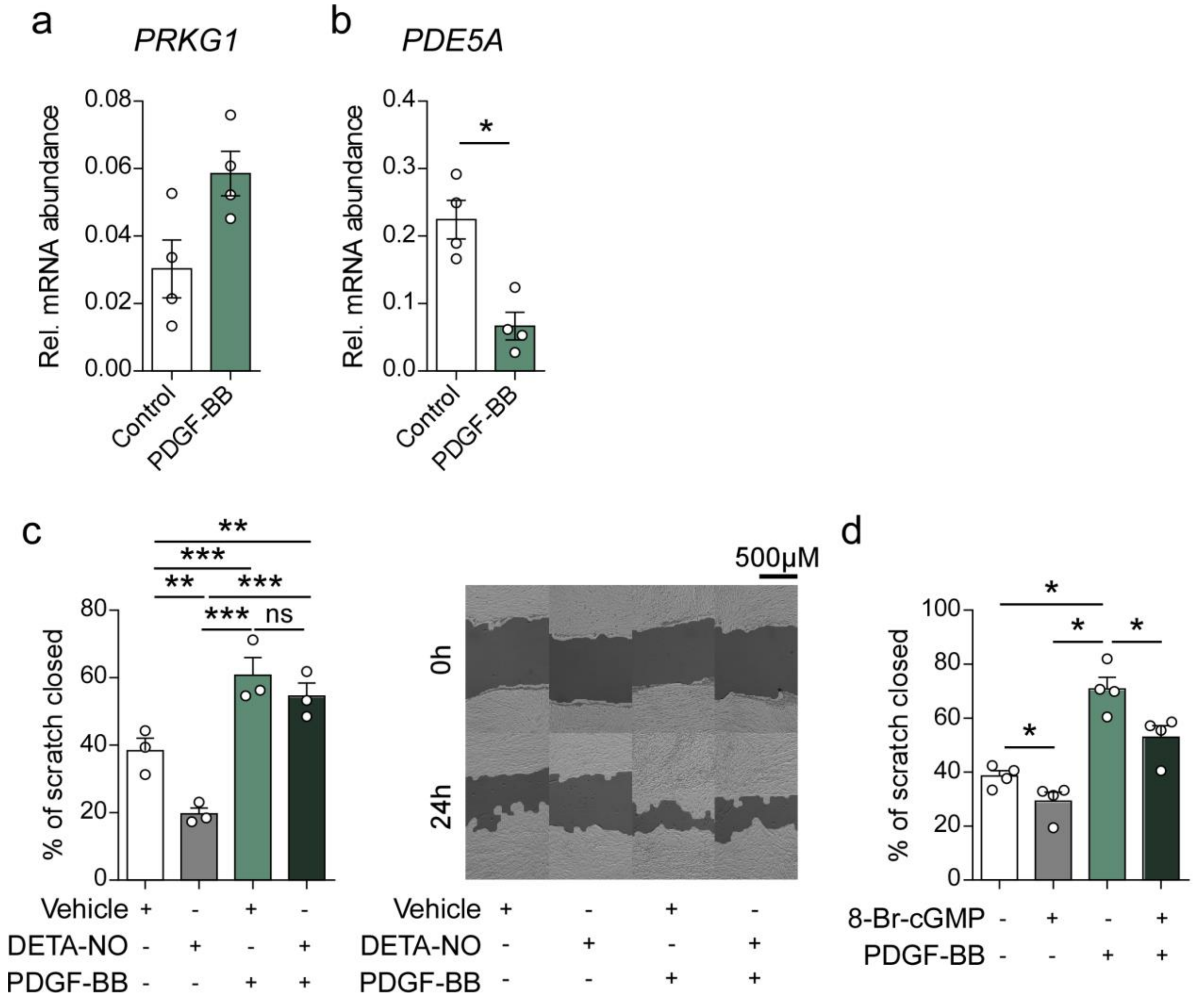

Supplementary Figure 1. Effect of PDGF on the cGMP pathway. a Expression of *PRKG1* mRNA in hASMCs after treatment with PDGF-BB; t-test; n=4. **b** Expression of *PDE5A* mRNA in hASMCs after treatment with PDGF-BB; t-test; n=4. **c** NO-induced (100 µM DETA-NO) inhibition of hASMC migration after treatment with PDGF-BB (scratch assay); ANOVA with Tukey's multiple comparisons test; n=3. Representative images from 3 replicates. **d** cGMP (100 µM 8-Br-cGMP) inhibition of hASMC migration after treatment with PDGF-BB; ANOVA with Tukey's multiple comparisons test; n=4. Bars indicate means ± SEM. *p<0.05.

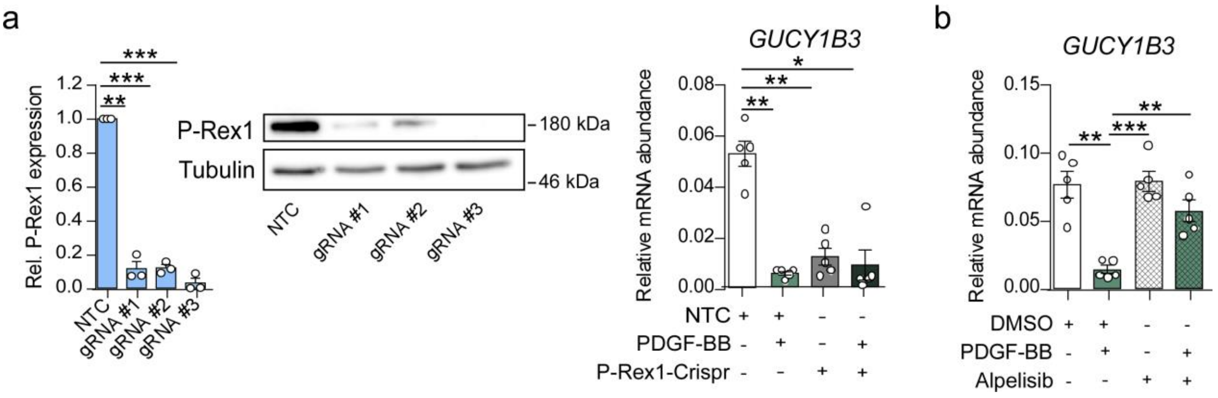

Supplementary Figure 2. sGCβ1 expression and P-Rex1 signaling in hASMCs. **a** Evaluation of three different gRNAs for P-Rex1 knock-down using CRISPR/Cas9 (left); n=3. qPCR analysis of GUCY1B3 mRNA expression in hASMCs transduced with CRISPR/Cas9 gRNA #3 (P-Rex1-Crispr) or non-targeting control (NTC) (right); ANOVA with Tukey's multiple comparisons test; n=5. **b** qPCR analysis of *GUCY1B3* expression in hASMCs after treatment with PDGF-BB with or without Alpelisib (20 µM); ANOVA with Tukey's multiple comparisons test; n=5. Bars indicate means ± SEM. *p<0.05, **p<0.01, ***p<0.001.

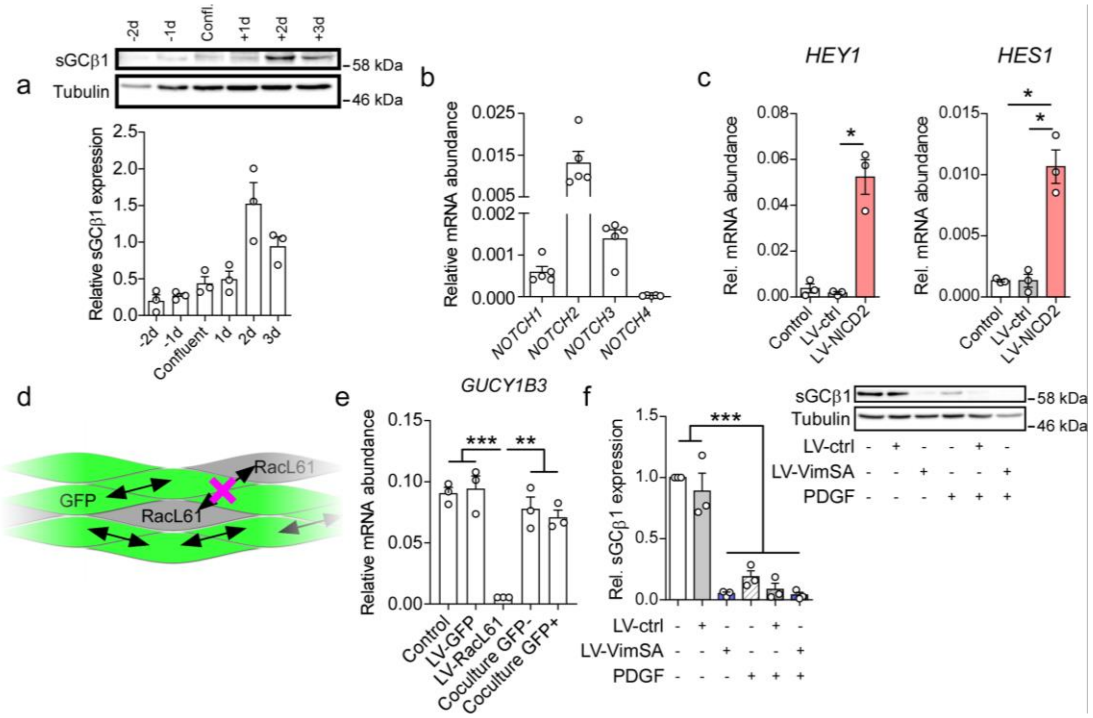

Supplementary Figure 3. sGCβ1 expression and Notch signaling in hASMCs. **a** Timecourse of sGCβ1 protein expression over increasing states of cell confluence; n=3. **b** Expression of the different Notch

isoforms in hASMCs; n=5. **c** Expression of *HES1* and *HEY1* mRNA in hASMCs after lentiviral transduction with N2ICD or control vector; ANOVA with Tukey's multiple comparisons test; n=3. **d** Schematic of co-culture model used to differentiate between signal-sending and signal-receiving cells in hASMCs expressing either GFP or RacL61. **e** qPCR analysis of *GUCY1B3* expression in co-cultured cells compared to cells expressing only GFP (LV-GFP) or RacL61 (LV-RacL61); ANOVA with Tukey's multiple comparisons test; n=3. **f** Western blot analysis of sGCß1 protein levels in hASMCs after lentiviral transduction with S4,6,7,8,9A-Vimentin (LV-VimSA) or control vector (LV-ctrl) with or without concomitant treatment with PDGF-BB; ANOVA with Tukey's multiple comparisons test; n=3. Bars indicate means ± SEM. *p<0.05, **p<0.01, ***p<0.001.

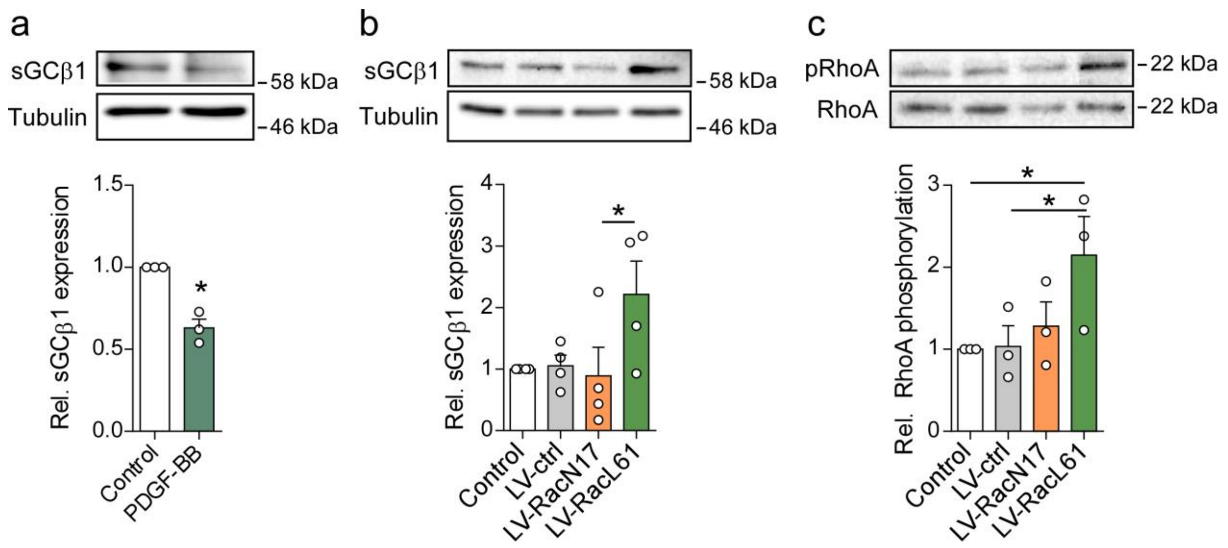

Supplementary Figure 4. sGC is differentially regulated by PDGF/Rac1 in human and murine VSMC.

a Western blot analysis of sGCß1 protein levels in mASMCs after treatment with 100ng/ml PDGF-BB; t-test; n=3. **b** Western blot analysis of sGCß1 protein levels in mASMCs after lentiviral transduction with RacN17, RacL61, or control vector; ANOVA with Tukey's multiple comparisons test; n=4. **c** Western blot analysis of RhoA phosphorylation after lentiviral transduction with RacN17, RacL61, or control vector; ANOVA with Tukey's multiple comparisons test; n=3. Bars indicate means ± SEM. *p<0.05.

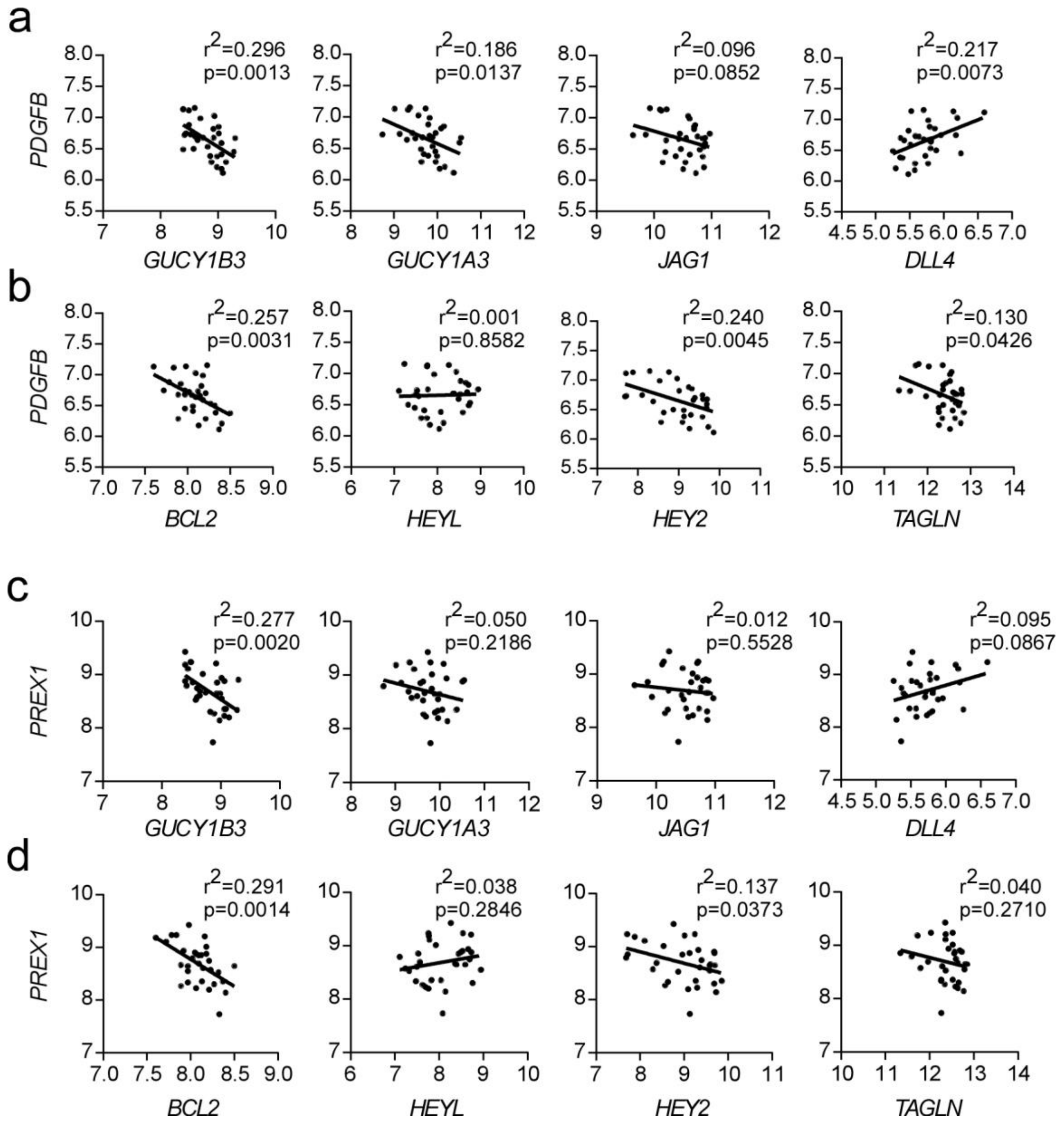

Supplementary Figure 5. PDGFB and PREX1 correlates with Notch ligand switching and negative sGC regulation in early stage human carotid artery atherosclerotic lesions. a Correlation of *PDGFB* with *GUCY1A3*, *GUCY1B3*, *JAG1*, and *DLL4* expression. **b** Correlation of *PDGFB* with *BCL2*, *HEYL*, *HEY2*, and *TAGLN* expression. **c** Correlation of *PREX1* with *GUCY1A3*, *GUCY1B3*, *JAG1*, and *DLL4* expression. **d** Correlation of *PREX1* with *BCL2*, *HEYL*, *HEY2*, and *TAGLN* expression. Sample data obtained from GEO dataset GSE43292. Correlation analysis was performed with Pearson's correlation test.

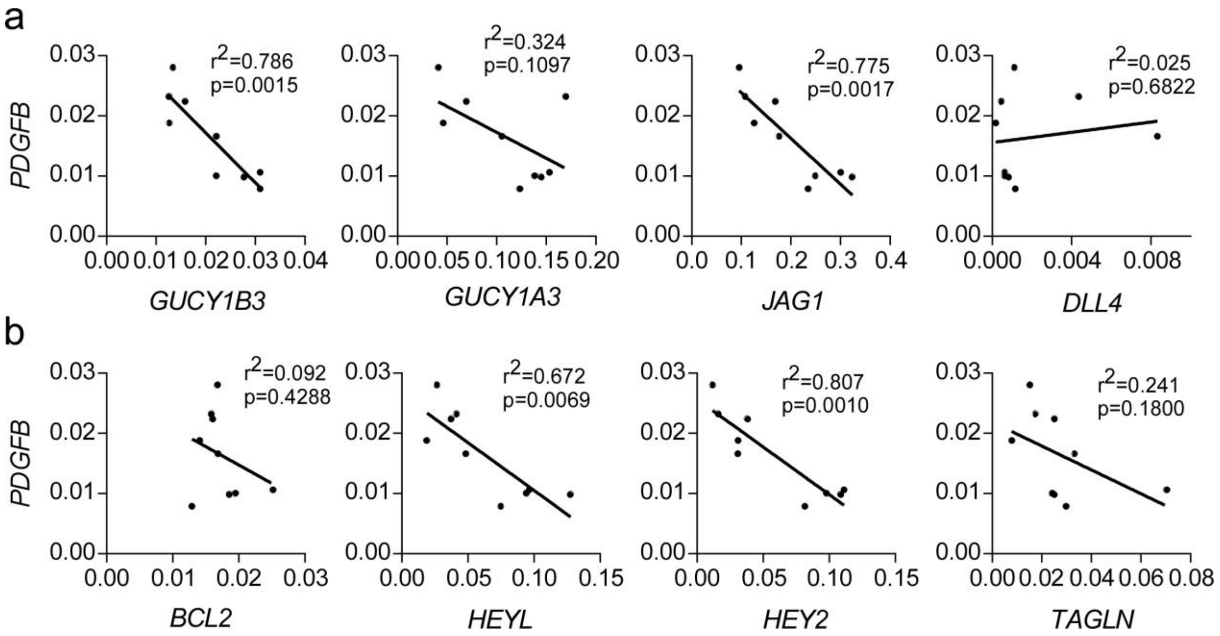

Supplementary Figure 6. RT-qPCR analysis shows correlation between PDGFB and Notch ligand switching as well as negative sGC regulation in early stage human carotid artery atherosclerotic lesions. a Correlation of *PDGFB* with *GUCY1A3*, *GUCY1B3*, *JAG1*, and *DLL4* expression. **b** Correlation of *PDGFB* with *BCL2*, *HEYL*, *HEY2*, and *TAGLN* expression. Correlation analysis was performed with Pearson's correlation test.

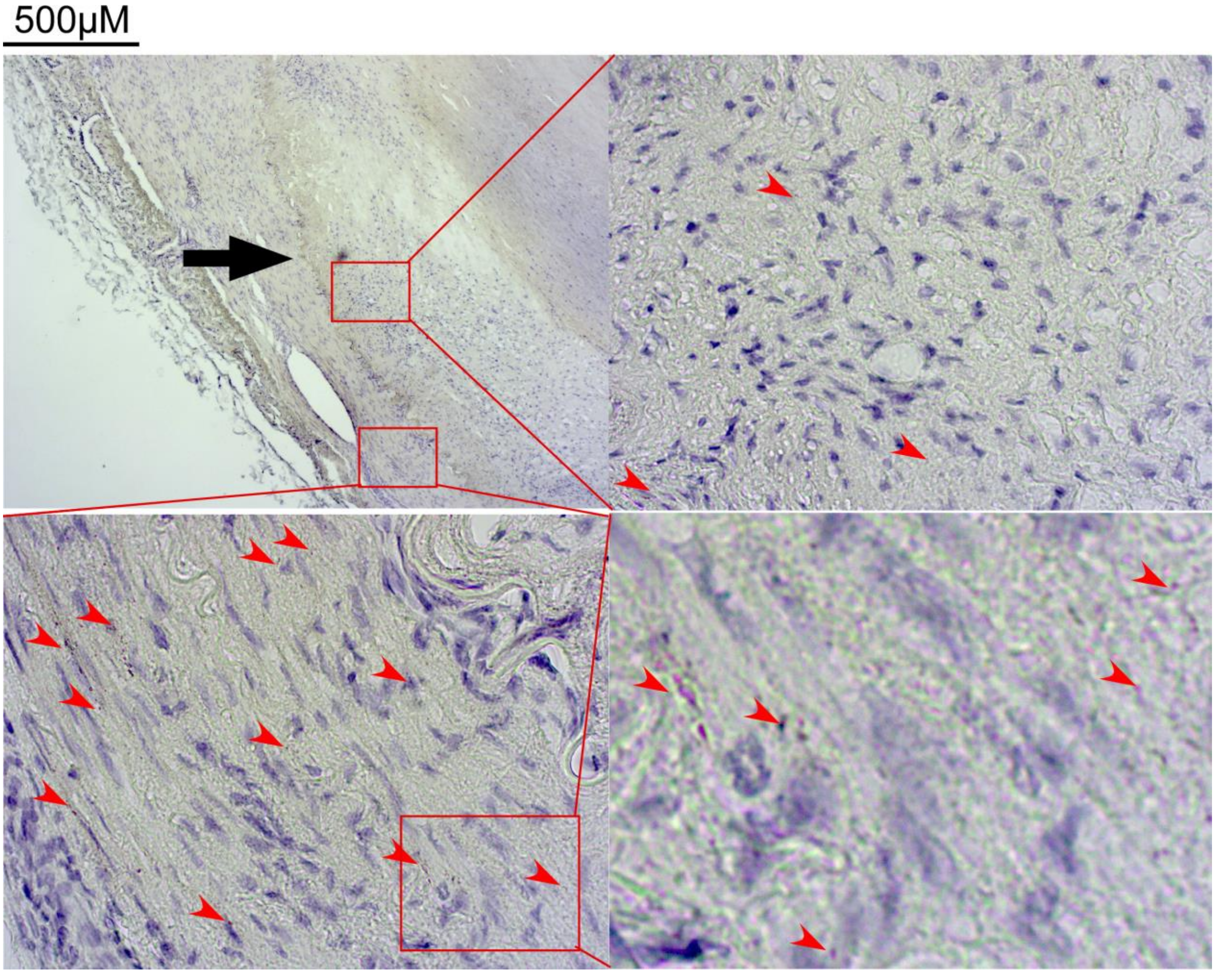

Supplementary Figure 7. RNAscope *in situ*-hybridization for *GUCY1B3* mRNA in PAD sections. Black arrow indicates internal elastic lamina. Red arrows show RNAscope punctate dots indicating *GUCY1B3* hybridization.

Figure 1b:

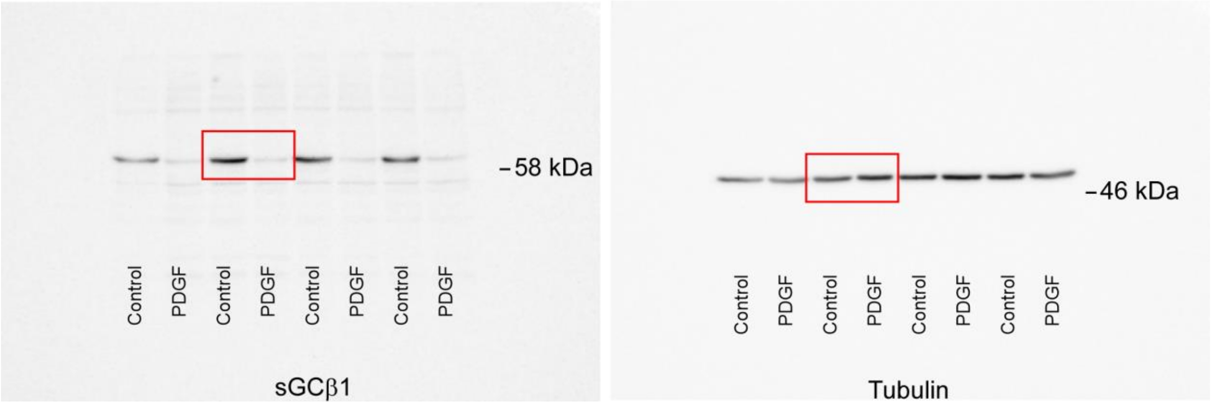

Figure 1c:

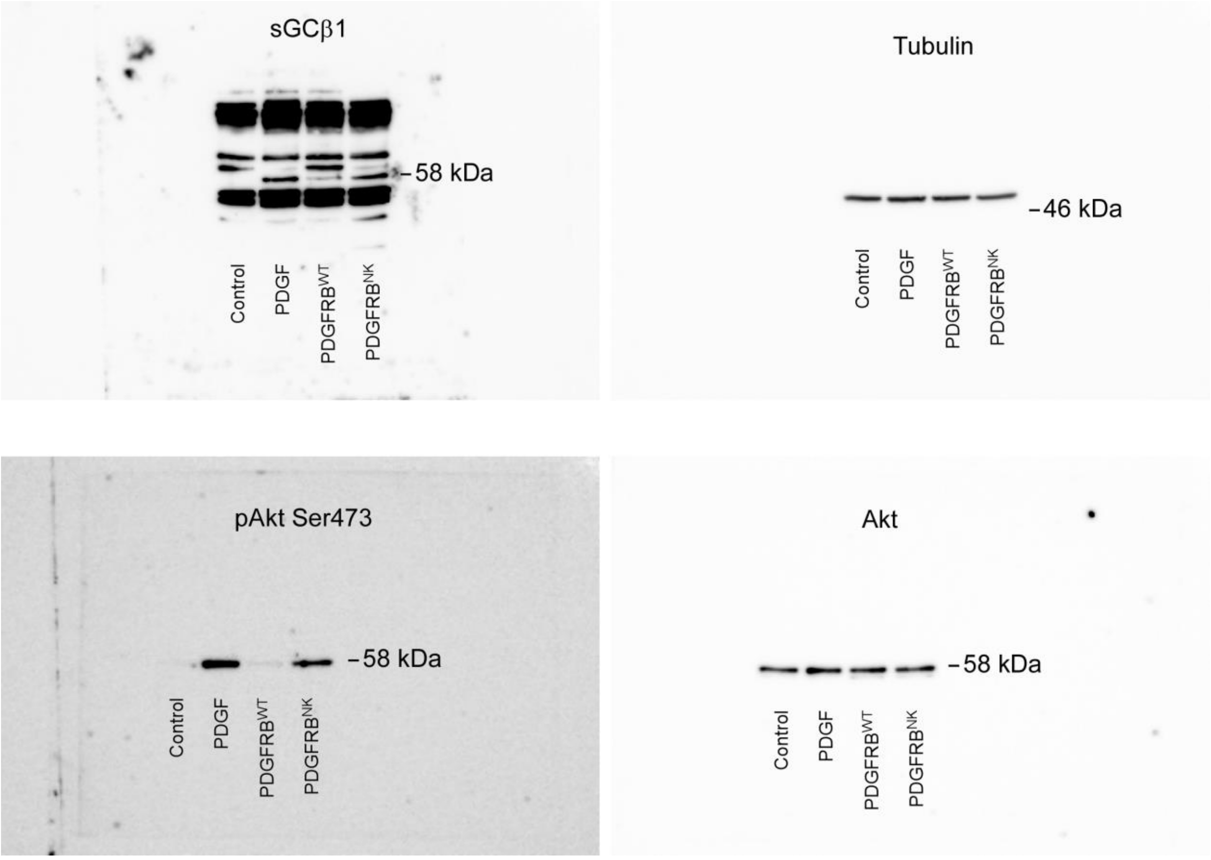

Figure 2a:

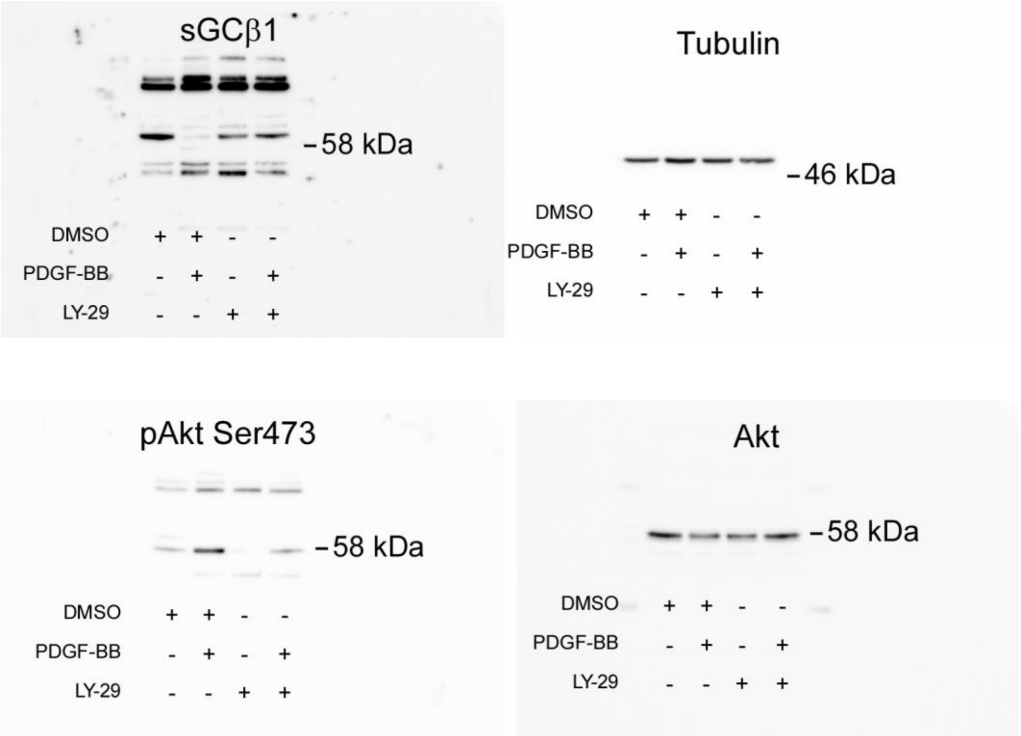

Figure 2b:

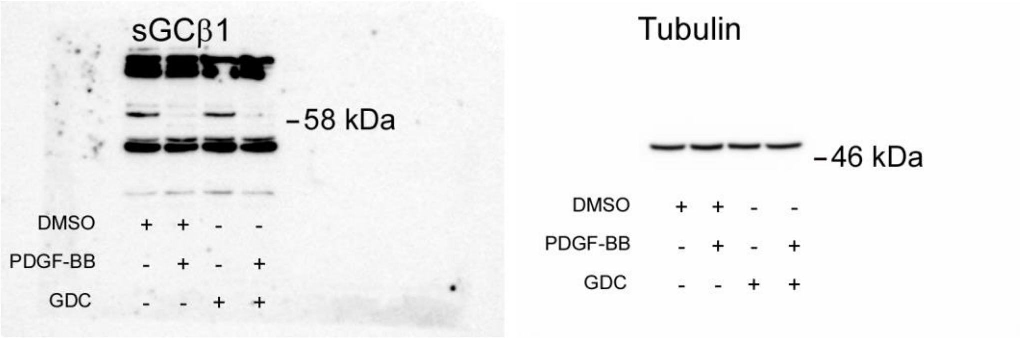

Figure 2c:

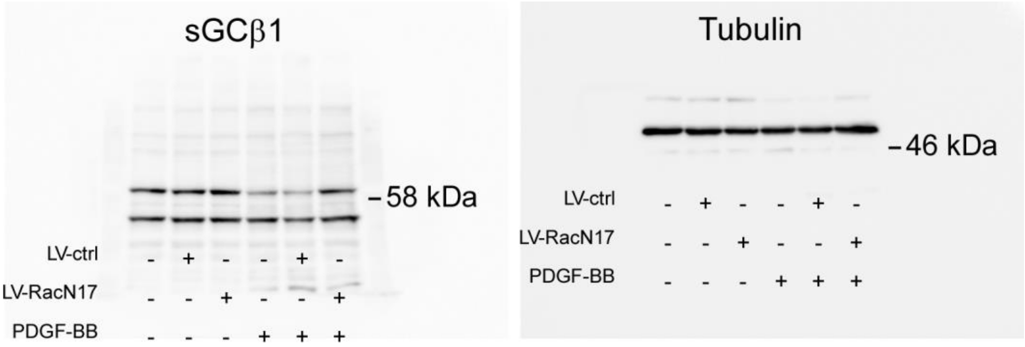

Figure 2d:

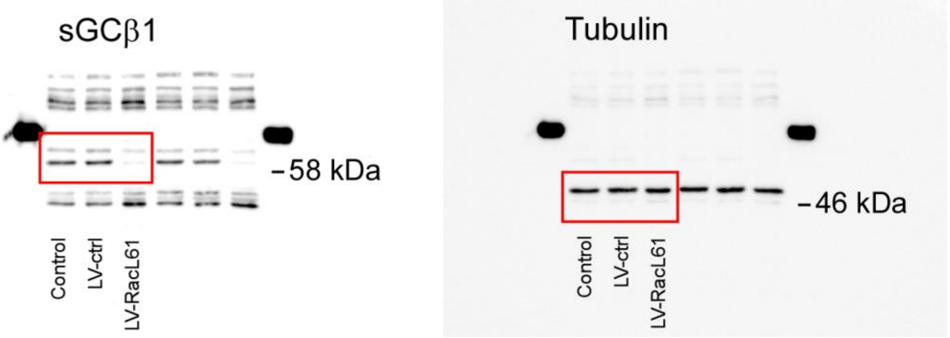

Figure 2f:

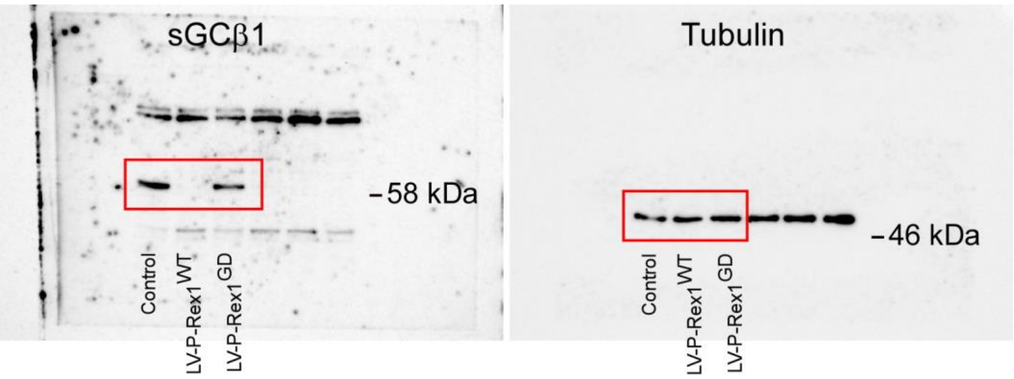

Figure 3a:

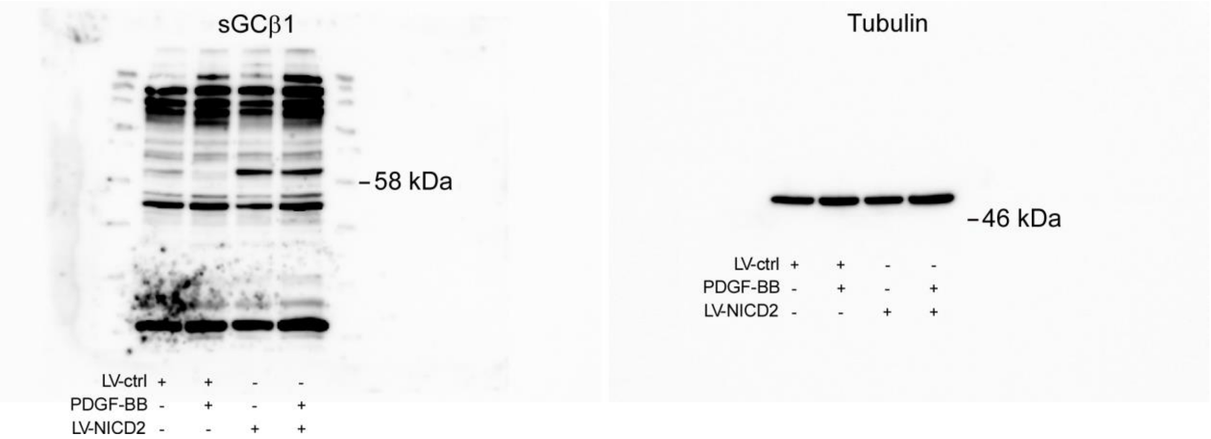

Figure 3f:

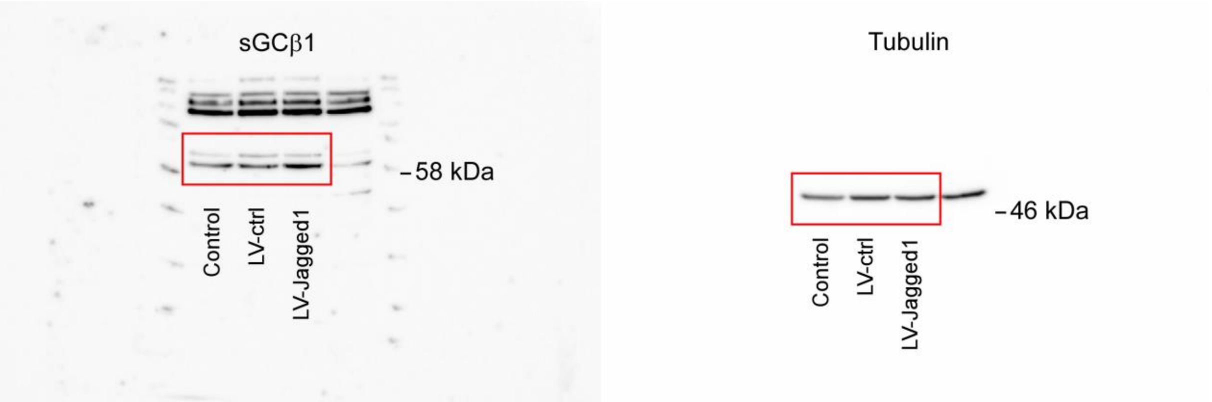

Figure 3h:

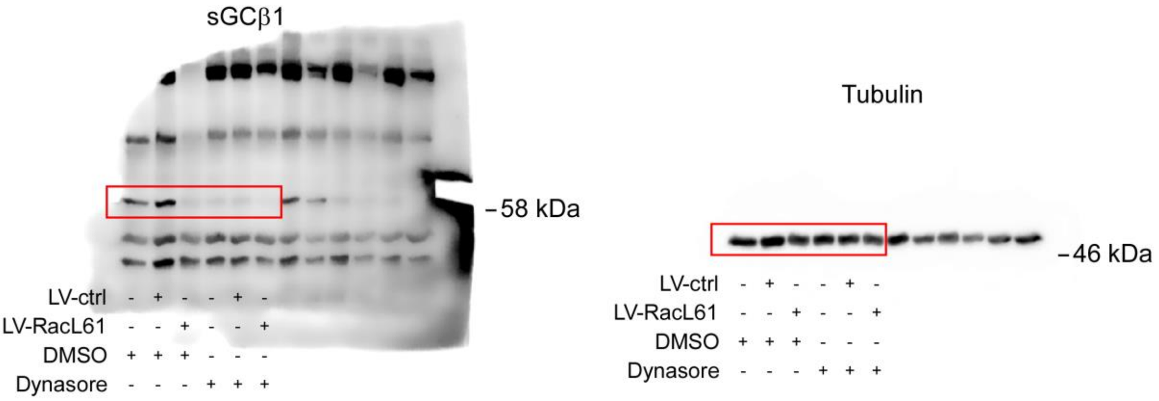

Figure 3j:

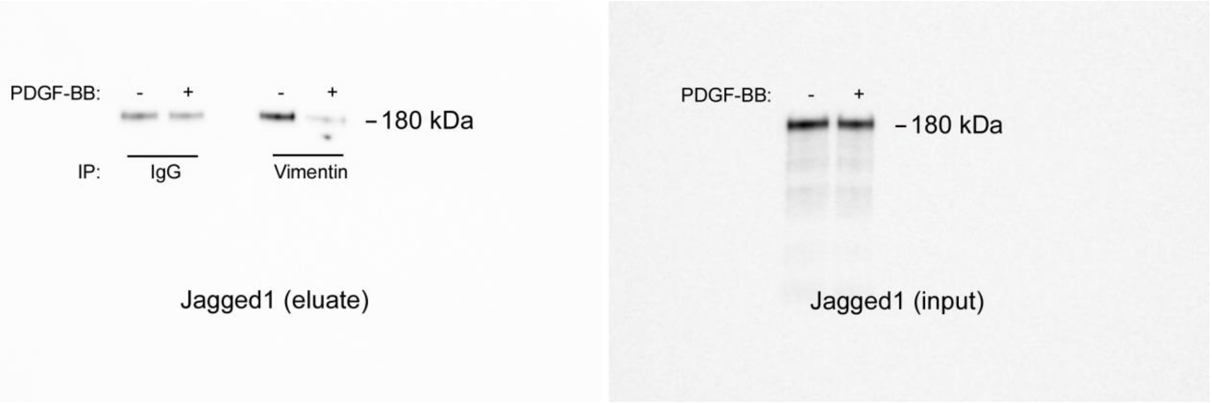

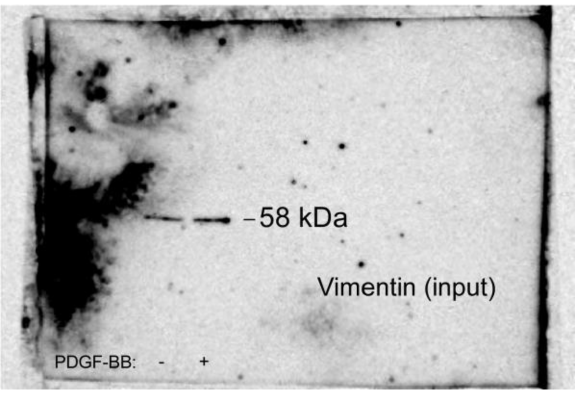

Figure S2a:

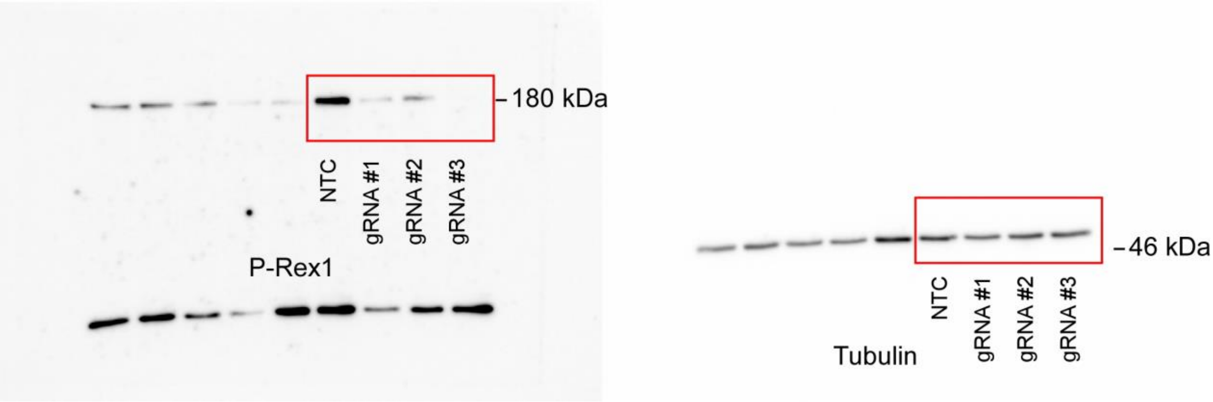

Figure S3a:

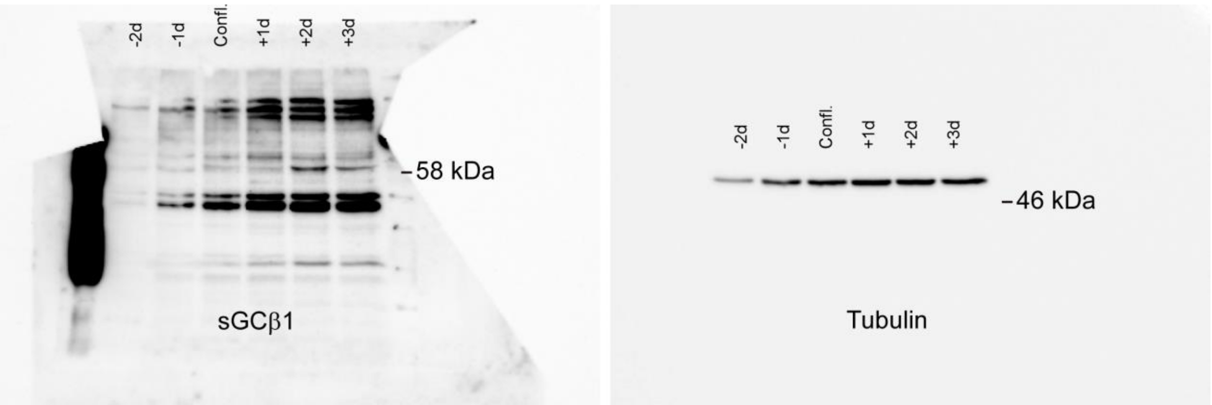

Figure S3f:

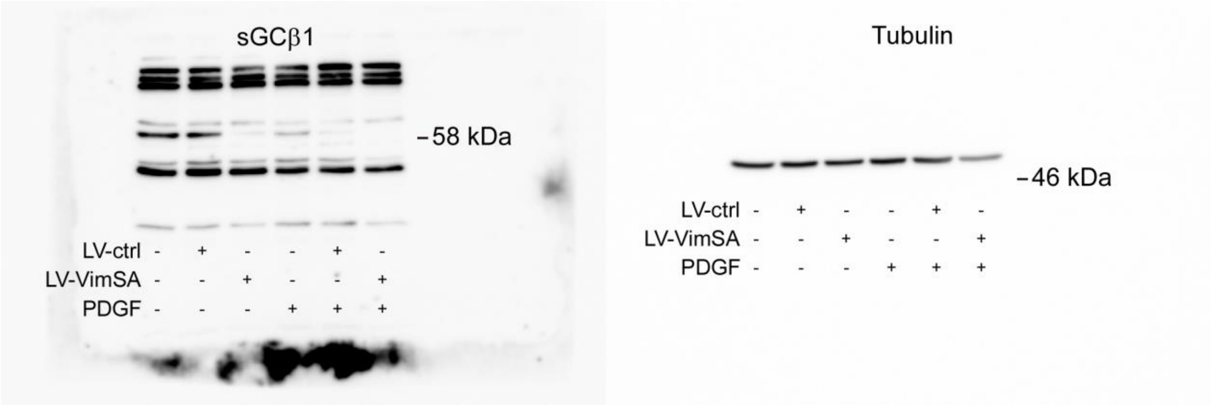

Figure S4a:

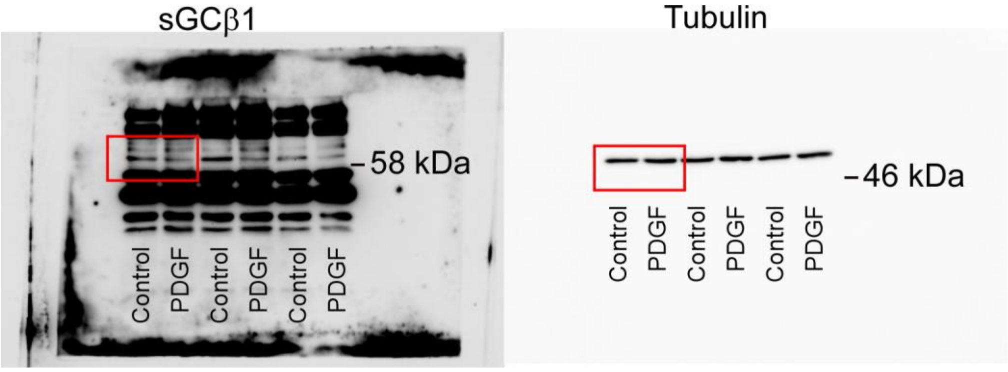

Figure S4b:

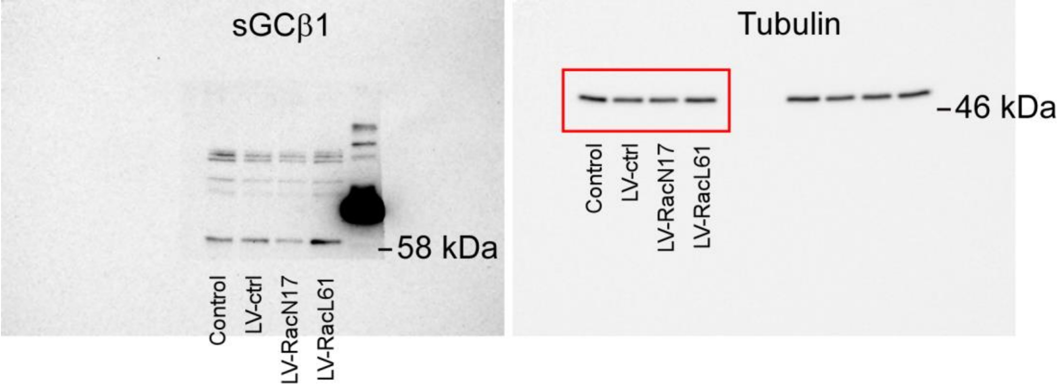

Figure S4c:

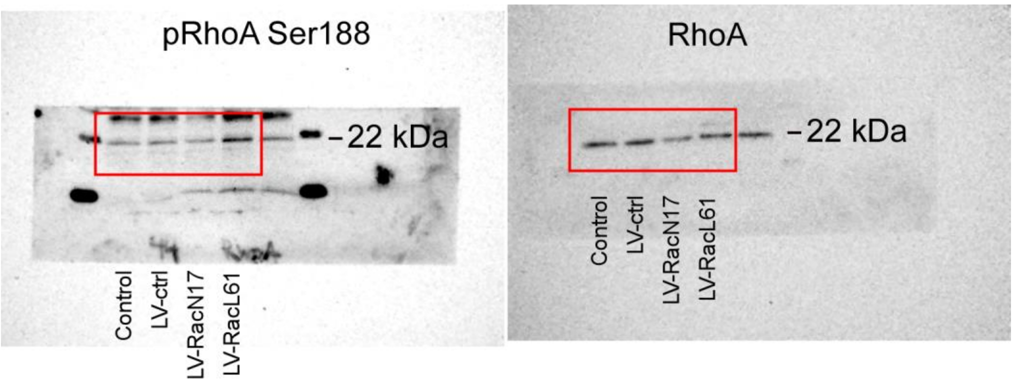

Supplementary Figure 8. Full, uncropped Western blots. In blots where samples (lanes) not represented in the original figures are present, the lanes represented in the original figures are marked with a red rectangle.

GUCY1A3

Species	NW score	Identity
Mus musculus	-1374	47%
Rattus norvegicus	-1167	47%
Oryctolagus cuniculus	-105	59%
Ovis aries	-1432	46%
Sus scrofa	-1307	47%
Bos Taurus	-1132	43%
Pan troglodytes	2843	98%

GUCY1B3

Species	NW score	Identity
Mus musculus	-1005	50%
Rattus norvegicus	-1039	50%
Oryctolagus cuniculus	-133	58%
Ovis aries	-117	58%
Sus scrofa	-39	59%
Bos Taurus	71	62%
Pan troglodytes	2915	99%

Supplementary Data 1. Comparison of GUCY1A3 and GUCY1B3 promoter regions in laboratory animals. Needleman-Wunsch alignment and sequence identities of GUCY1A3 and GUCY1B3 promoter regions (-1500bp) in different laboratory animals compared to human.
