# Supplementary material for: PDGF regulates guanylate cyclase expression and cGMP signaling in vascular smooth muscle
Source: Commun Biol. 2022 Mar 3;5:197. doi: 10.1038/s42003-022-03140-2 (PMC8894477; doi:10.1038/s42003-022-03140-2)
Supplement: Supplementary file 2 — Description of Additional Supplementary Files [file 42003_2022_3140_MOESM2_ESM.pdf]

Description of Additional Supplementary Files

File name: Supplementary Data 1

Description: All source data underlying the graphs in the main figures.
