# Supplementary material for: PDGF regulates guanylate cyclase expression and cGMP signaling in vascular smooth muscle
Source: Commun Biol. 2022 Mar 3;5:197. doi: 10.1038/s42003-022-03140-2 (PMC8894477; doi:10.1038/s42003-022-03140-2)
Supplement: Supplementary file 4 — Reporting Summary [file 42003_2022_3140_MOESM4_ESM.pdf]

nature research

Corresponding author(s): Staffan Hildebrand, Alexander Pfeifer

Last updated by author(s): Dec 14, 2021

Reporting Summary

Nature Research wishes to improve the reproducibility of the work that we publish. This form provides structure for consistency and transparency in reporting. For further information on Nature Research policies, see our Editorial Policies and the Editorial Policy Checklist.

Statistics

For all statistical analyses, confirm that the following items are present in the figure legend, table legend, main text, or Methods section.

n/a	Confirmed	
☐	☒	The exact sample size (*n*) for each experimental group/condition, given as a discrete number and unit of measurement
☐	☒	A statement on whether measurements were taken from distinct samples or whether the same sample was measured repeatedly
☐	☒	The statistical test(s) used AND whether they are one- or two-sided<br>*Only common tests should be described solely by name; describe more complex techniques in the Methods section.*
☒	☐	A description of all covariates tested
☐	☒	A description of any assumptions or corrections, such as tests of normality and adjustment for multiple comparisons
☐	☒	A full description of the statistical parameters including central tendency (e.g. means) or other basic estimates (e.g. regression coefficient) AND variation (e.g. standard deviation) or associated estimates of uncertainty (e.g. confidence intervals)
☒	☐	For null hypothesis testing, the test statistic (e.g. F, t, r) with confidence intervals, effect sizes, degrees of freedom and P value noted<br>*Give P values as exact values whenever suitable.*
☒	☐	For Bayesian analysis, information on the choice of priors and Markov chain Monte Carlo settings
☒	☐	For hierarchical and complex designs, identification of the appropriate level for tests and full reporting of outcomes
☒	☐	Estimates of effect sizes (e.g. Cohen's d, Pearson's r), indicating how they were calculated

Our web collection on statistics for biologists contains articles on many of the points above.

Software and code

Policy information about availability of computer code

Data collection	ABI StepOne Software 2.3, LAS 4000 Control Software, ZEN 2.3
Data analysis	ImageJ 1.48v, ABI StepOne Software 2.3, Microsoft Excel 2007, FlowJo v10, Graphpad Prism 6

For manuscripts utilizing custom algorithms or software that are central to the research but not yet described in published literature, software must be made available to editors and reviewers. We strongly encourage code deposition in a community repository (e.g. GitHub). See the Nature Research guidelines for submitting code & software for further information.

Data

Policy information about availability of data

All manuscripts must include a data availability statement. This statement should provide the following information, where applicable:

- Accession codes, unique identifiers, or web links for publicly available datasets
- A list of figures that have associated raw data
- A description of any restrictions on data availability

The data that support the findings of this study are available from the corresponding author upon reasonable request.

Field-specific reporting

Life sciences study design

All studies must disclose on these points even when the disclosure is negative.

Sample size	n/a
Data exclusions	No data was excluded
Replication	Every experiment was repeated a minimum of three times to ensure reproducibility
Randomization	Due to the nature of the expreiments, randomization of the samples was not applicable
Blinding	The investigators were not blinded to group allocation in this study

Reporting for specific materials, systems and methods

We require information from authors about some types of materials, experimental systems and methods used in many studies. Here, indicate whether each material, system or method listed is relevant to your study. If you are not sure if a list item applies to your research, read the appropriate section before selecting a response.

Materials & experimental systems

n/a	Involved in the study	
☐	☒	Antibodies
☒	☐	Eukaryotic cell lines
☒	☐	Palaeontology and archaeology
☒	☐	Animals and other organisms
☐	☒	Human research participants
☒	☐	Clinical data
☒	☐	Dual use research of concern

Methods

n/a	Involved in the study	
☒	☐	ChIP-seq
☒	☐	Flow cytometry
☒	☐	MRI-based neuroimaging

Antibodies

Antibodies used

Akt Cell Signaling, Danvers, USA 9272S 1:1000
GAPDH Cell Signaling, Danvers, USA 2118S 1:1000
Goat anti-Rabbit IgG AF-555 ThermoFischer Scientific, Darmstadt A-21428 1:500
IgG isotype control Cell Signaling, Danvers, USA 3900S 0.1 µg (IP)
Jagged-1 Cell Signaling, Danvers, USA 70109S 1:1000
Anti-Mouse-HRP Cell Signaling, Danvers, USA 7076S 1:10000
pAkt Serine 473 Cell Signaling, Danvers, USA 9271S 1:1000
pRhoA Serine 188 Santa Cruz, Santa Cruz, USA Sc-32954 1:1000
Anti-Rabbit-HRP Cell Signaling, Danvers, USA 7074S 1:5000
RhoA Santa Cruz, Santa Cruz, USA Sc-418 1:1000
sGCβ1 Sigma-Aldrich, Munich, Germany G4405 1:1000 (WB)
1:100 (Immunostaining)
Tubulin Dianova, Hamburg, Germany DLN-09993 1:1000
Vimentin Cell Signaling, Danvers, USA 5741S 1:1000 (WB)
1:100 (Immunostaining)
0.1 µg (IP)
P-Rex1 Cell Signaling, Danvers, USA 13168S 1:1000 (WB)

Validation

Validation was done by over-expression or knock-down experiments where possible

Human research participants

Policy information about studies involving human research participants

Population characteristics	Consenting patients undergoing coronary bypass surgery at the Klinik und Poliklinik für Herzchirurgie, University Clinic Bonn, Bonn, Germany, were enrolled in the study.
Recruitment	Patients were recruited prior to undergoing coronary bypass sugery. No potential bias in patient recruitment was present.
Ethics oversight	All experiments using patient IMA samples were approved by the Ethics Commission at the University Clinic Bonn, Bonn, Germany.

Note that full information on the approval of the study protocol must also be provided in the manuscript.
